# A Low-Cost, Accurate, and Easily-Worn E-Skin and IMU Hand Kinematic Measurement System

**DOI:** 10.3390/s26154795

**Published:** 2026-07-28

**Authors:** Tomas Oppenheim, Hanna Schlegel, Phil Yuantai Xie, Zeyad Khokhar, Preeya Khanna

**Affiliations:** 1 Department of Mechanical Engineering, Cal Poly Maritime Academy, Vallejo, CA 94590, USA; toppenheim@csum.edu; 2 Department of Neuroscience, University of California, Berkeley, CA 94720, USA; 3 Department of Electrical Engineering and Computer Sciences, University of California, Berkeley, CA 94720, USA; hanna_schlegel@berkeley.edu (H.S.); phil.xie@berkeley.edu (P.Y.X.); 4 Department of Bioengineering, University of California, Berkeley, CA 94720, USA; zeyadkhokhar@berkeley.edu

**Keywords:** wearable, kinematics, rehabilitation, hand

## Abstract

**Highlights:**

**What are the main findings?**

- Developed an accurate, multimodal wearable system for hand and wrist kinematic tracking that combines soft stretchable e-skin finger bend sensors with IMUs.
- Demonstrated usability, robustness, and affordability, with naïve users independently wearing the full system in 5 min, a demonstration of multi-hour out-of-lab hand tracking, and a low device cost of $111 (estimated $55 at scale).

**What are the implications of the main findings?**

- The rapid, independent user setup establishes the feasibility of deploying this wearable system for out-of-lab neurorehabilitation applications.
- The low unit cost and economic scalability enable widespread, accessible, and continuous long-term kinematic monitoring in real-world settings.

**Abstract:**

Stroke and other neurological injuries impair hand function. Although rehabilitation therapists encourage reintegration of the affected hand into daily activities, there are few tools that can be worn during everyday life that provide quantitative feedback on how much or how effectively the hand is used. Wearable sensors that can accurately track hand movements and are easily applied and removed can present intuitive feedback that could motivate hand use similarly to how pedometers encourage walking. While tracking all the hand and finger joints is needed for scientific studies, under-sensorization, or using fewer sensors than required for tracking all degrees of freedom, may suffice for providing users feedback about hand use in everyday life. Further, it may enable a wearable device to be more easily donned and doffed, more power efficient, and more cost efficient. Here we develop a low-cost, multi-sensor, wireless wearable system for tracking selected hand and wrist movements during everyday life. The system includes fabricated soft, stretchable “e-skin” bend sensors and off-the-shelf inertial measurement units (IMUs) that accurately measure finger bend angles and wrist movements. The system also includes an application and removal protocol that enabled naïve unimpaired participants to apply and remove the system in ~5 min and ~4 min, respectively. The system cost was $111 per device, with prices falling to an estimate of $55 when manufactured at scale. This hand wearable demonstrates accurate kinematic tracking and user-friendly donning/doffing workflows for unimpaired participants, making it a promising platform for everyday hand tracking. Future work will extend this platform to the movement-impaired population for neurorehabilitation applications.

## 1. Introduction

Stroke and other neurological injuries frequently impair hand function, and despite structured physical therapy and other interventions, most individuals are left with long-term upper-limb disability [1]. Some studies show that high-intensity, high-dose rehabilitation interventions on the order of 25–30 h per week over multiple weeks are associated with significantly improved motor outcomes [2,3]. However, delivering such high dosages in real-world settings where time, cost, and therapist availability limit the scalability of these interventions remains challenging.

One strategy to increase the rehabilitation dose without increasing the therapist time is to encourage use of the paretic limb during activities of daily living outside of the clinical setting. Constraint-induced movement therapy (CIMT) does this by constraining the non-paretic limb, thereby forcing use of the impaired hand, and has excellent outcomes [4,5]. While CIMT can be effective, its use is limited: many patients do not meet the baseline motor function required to participate in CIMT since it requires that nearly all daily living tasks can be performed with the paretic hand. There are not currently ways to extend this therapy to more severely impaired people who do not qualify.

A limitation of all rehabilitation paradigms is the lack of objective measurement of out-of-clinic real-world hand use. Having such a measurement derived from hand and wrist joint angle kinematics and displaying feedback to patients could serve to encourage more hand use in daily life. Further, clinicians could monitor hand-use and hand kinematics to verify that patients are completing prescribed physical therapy exercises to improve adherence [6], effectively increasing the dosage of physical therapy. Researchers developing new therapeutic interventions could monitor out-of-lab hand use to study how interventions affect hand-use in the real-world, and account for different effects of their interventions in different patients. Beyond hand use, a comfortable hand wearable system could also serve as a platform for developing at-home biofeedback therapeutic interventions [7,8], and studying questions about how well movements practiced in physical therapy transfer to the patient’s everyday life.

In this study, we develop a low-cost, accurate, easily worn e-skin and IMU under-sensorized hand kinematics measurement system. Under-sensorization refers to sensorizing only a subset of the degrees of freedom needed for complete hand tracking. While many hand-tracking technologies have been developed, most are in the form of a glove that can be difficult for people with movement impairments to don/doff and impedes sensation [9,10,11,12,13], or are worn on the back of the hand, leaving sensation intact, but are bulky and impractical for users to wear on their own [14,15,16,17]. Other approaches to tracking hand movements use cameras and computer vision pose-estimation algorithms [18,19,20,21], though developing a battery-powered wireless wearable that can capture camera frames and run pose-estimation algorithms remains a challenge. Most recently, wrist-mounted ultrasound imaging has emerged as a promising solution, though reliant on complex models to map sensor measurements to joint angles in a way that may not generalize to movement-impaired people [22]. Here, we use two types of sensors: (1) in-lab fabricated thin, flexible silicone “e-skin” liquid metal bend sensors and (2) inertial measurement units (IMUs) with on-board sensor fusion to measure finger and wrist bend angles, respectively. E-skin sensor measurements are digitized by a custom data acquisition board, and these measurements along with IMU measurements are streamed out to a nearby computer via Bluetooth. Beyond developing the wearable system, we have also created a user-friendly application and removal protocol. We conducted a user study that characterized the accuracy of the wearable system, as well as the ease of application and removal and comfort of the system.

Overall, we have created a low-cost, accurate, easily worn e-skin and IMU under-sensorized hand kinematics measurement system that we believe can serve as a starting point towards giving patients and clinicians feedback about in-the-wild hand usage.

## 2. Materials and Methods

### 2.1. Strain Sensor Fabrication

**Figure 1:**
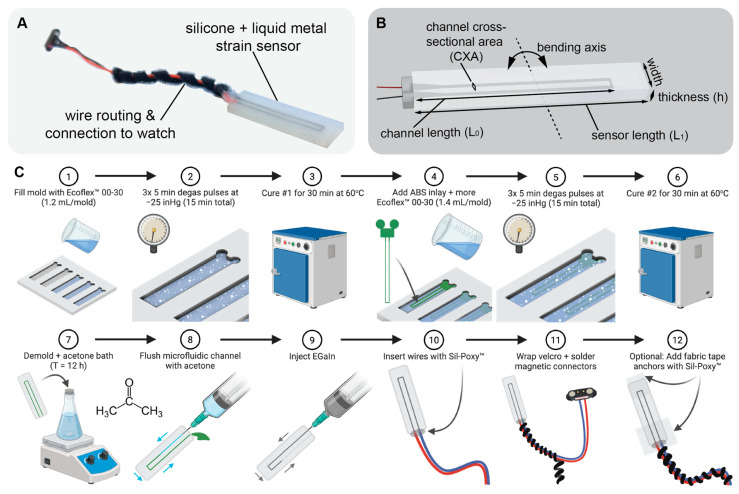
Liquid-metal strain sensor and fabrication workflow. (**A**) Assembled sensor showing wire routing and watch connector. (**B**) Sensor geometry with liquid-metal channel cross-sectional area (CXA), channel length (L_0_), sensor length (L_1_), width, thickness, and bending axis. (**C**) Stepwise fabrication: (1) Fill mold with Ecoflex 00-30 (1.2 mL/mold). (2) Conduct 3 × 5 min degas pulses at −25 inHg (15 min total). (3) Cure for 30 min at 60 °C. (4) Place ABS sacrificial inlay with alignment ears and add additional Ecoflex 00-30 (1.4 mL/mold). (5) Conduct 3 × 5 min degas pulses at −25 inHg (15 min total). (6) Cure for 30 min at 60 °C. (7) Demold, trim alignment ears, and soak in acetone (~12 h) to dissolve ABS. (8) Flush microchannel with acetone. (9) Inject eutectic gallium–indium (EGaIn; conductive liquid metal) to fill channel. (10) Insert leads and insulate ports with Sil-Poxy. (11) Add wire management (Velcro wrap) and solder magnetic connectors. (12) Optional: Bond fabric-tape anchors with Sil-Poxy. Partially created in BioRender. Schlegel, H. (2026). https://biorender.com/cyy7xqe (accessed on 19 June 2026).

Silicone microfluidic strain sensors with liquid-metal channels were fabricated using a two-step casting process with a sacrificial ABS inlay inspired by [23] (Figure 1C). Custom molds defining the sensor outline and microchannel alignment features were first filled with Ecoflex™ 00-30 silicone rubber (1.2 mL per mold; Smooth-On, Macungie, PA, USA). The molds were placed in a vacuum chamber and degassed using three short vacuum pulses: the chamber pressure was reduced to –25 inHg for 5 min, then returned to atmospheric pressure, and this sequence was repeated three times (for a total vacuum hold time of 15 min). The base layer was then cured for 30 min at 60 °C.

After the first cure, a sacrificial ABS inlay with alignment ears was positioned in each mold to define the microchannel. Additional Ecoflex™ 00-30 (1.4 mL per mold) was poured to fully encapsulate the inlay, followed by the same pulsed degassing procedure (three cycles of –25 inHg for 5 min with returns to atmospheric pressure between pulses for a total vacuum hold time of 15 min) and a 30 min cure at 60 °C. The cured sensors were demolded, the alignment ears were trimmed, and sensors were immersed in an acetone bath for ~12 h to dissolve the ABS, leaving a hollow microfluidic channel. The channels were then flushed with fresh acetone and allowed to dry at room temperature. To form the conductive element, the microchannel was filled with eutectic gallium–indium (EGaIn; RotoMetals, San Leandro, CA, USA) by injection from one port until liquid metal emerged from the opposite port. Two 28 AWG insulated wire leads (Fermerry, Shenzhen, China) were inserted into the ports and secured with a small amount of Sil-Poxy™ silicone adhesive (Smooth-On, Macungie, PA, USA), which also sealed and mechanically reinforced the interfaces. See Supplemental Methods “Strain sensor fabrication post-processing” for details on how the wire leads and mounting pads were routed and secured to the strain sensor and rest of the system.

Completed sensors measured approximately 43 mm sensor length × 11 mm sensor width × 3 mm sensor thickness (Figure 1B), weighed ~3 g, and exhibited an initial channel resistance of ~0.7 Ω at rest.

### 2.2. Strain Sensor Physics Model

The normalized resistance change of the liquid-metal microchannel as a function of mechanical axial strain followed the expression [24].

(1) $$\frac{\Delta R}{R_{0}}=\frac{\rho L_{0}\epsilon_{xx}}{\left(CXA\right)R_{0}}\left[\frac{8-\epsilon_{xx}}{{\left(2-\epsilon_{xx}\right)}^{2}}\right]$$

where ΔR is the change in electrical resistance, $R_{0}$ is the initial electrical resistance at rest, ρ is the electrical resistivity of the EGaIn liquid metal (29.4 × 10^−8^ ohm-m), *L*_0_ is the liquid-metal channel length (Figure 1B), *CXA* is the channel cross-sectional area of the microfluid channel (Figure 1B), and $\epsilon_{xx}$ is the mechanical axial strain. 

For bending over a curved knuckle, the mechanical axial strain followed the expression:

(2) $$\epsilon_{xx}=\frac{\Delta L}{L_{1}}=\frac{\left(R_{knuckle}+h\right)\theta }{L_{1}}$$

where $R_{knuckle}$ is the radius of the knuckle (in the benchtop setup this was a 3D printed knuckle with a radius of 6.3 mm), h is the sensor thickness (Figure 1B), θ is the bend angle, and $L_{1}$ is the initial sensor length at rest (Figure 1B).

### 2.3. Strain Sensor Characterization on Benchtop

**Figure 2:**
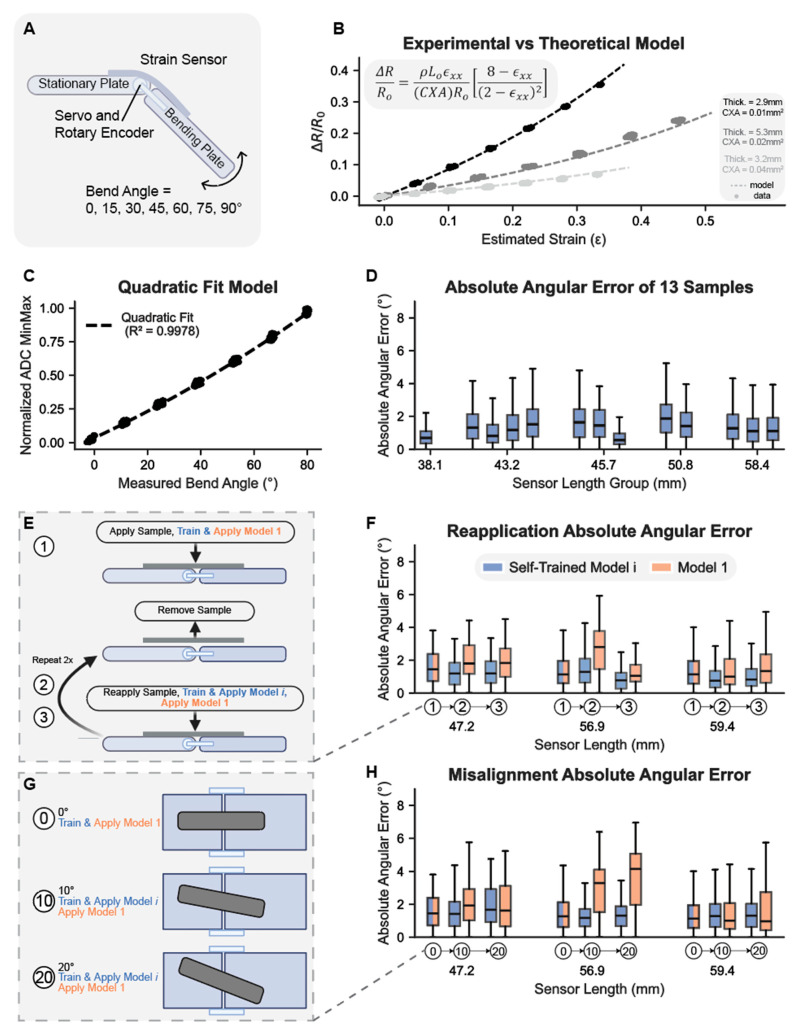
Benchtop testing of strain samples using an autobender. (**A**) Schematic of the autobender. The strain sensor was attached to both the fixed and moving plates using Velcro. As the moving plate was actuated by a servo motor, the sensor bent over a curved radius in 15 deg increments from 0 deg to 90 deg and back, completing five full cycles. The ground truth bend angle was measured using a rotary encoder mounted coaxially with the servo motor’s axis of rotation. (**B**) Change in resistance ($\Delta R/R_{0})$ versus estimated mechanical strain ($\epsilon_{XX}$). Experimental data (points) are compared against the predicted theoretical model (dashed line) for three different cross-sectional areas (CXA). The inset equation shows the theoretical relationship between the normalized resistance and mechanical strain. In the legend, “thick” corresponds to the thickness of the sample. Agreement between the experimental measurements and the theoretical model was quantified using variance accounted for (VAF), which was 0.999, 0.994, and 0.993 for the three samples. (**C**) Normalized sensor values versus bend angle and the quadratic fit. The data shown are the same experimental data as the black curve in (**B**). (**D**) Absolute angular error box plots for 13 different sensor samples grouped into five sensor-length categories. A one-way ANOVA did not detect a statistically significant effect of sensor length on mean absolute error (F(4, 8) = 1.41, *p* = 0.31, $\omega^{2}$ = 0.11). (**E**) Schematic illustrating the reapplication process. Numbers indicate the application numbers: (1) apply sensor for the first time, and train Model 1, (2–3) apply sensor for the second and third times and train “Self-Trained Model i” models, and apply self-trained models and Model 1. (**F**) Reapplication absolute angular error box plots for three different samples in the sensor reapplication experiments. The split blue/orange boxes correspond to application (1) in (**E**). Blue-only boxes correspond to errors from self-trained models in reapplication steps 2–3, and orange-only boxes correspond to errors obtained by applying the first-application calibration model (Model 1). When MAEs were calculated by averaging within each sample across reapplications, Model 1 produced significantly higher errors than the corresponding self-trained models (paired *t*-test, t(2) = 6.72, *p* = 0.02). The estimated mean paired difference was 0.64° (95% CI [0.23°, 1.06°], Cohen’s d = 3.83, Hedges’ g = 2.19). No statistically significant difference was detected between the second and third reapplications when using Model 1 (paired *t*-test, t(2) = −0.82, *p* = 0.50). The estimated mean difference was −0.43° (95% CI [−2.66°, 1.81°], Cohen’s d = −0.47, Hedges’ g = −0.27). (**G**) Schematic of misalignment angular error testing on the autobender. The same sample was intentionally misaligned by 10 deg and 20 deg to evaluate the resulting angular error. (**H**) Misalignment absolute angular error box plots for three different samples. Split blue/orange boxes correspond to the 0° aligned condition. Blue-only boxes represent errors from self-trained models, while orange-only boxes represent errors obtained by applying the 0° calibration model (“Model 1”) to the misaligned data. No statistically significant difference was detected between errors derived from self-trained models and those obtained using Model 1 (paired *t*-test, t(2) = 1.29, *p* = 0.33). The estimated mean difference was 1.67° (95% CI [−3.79°, 7.13°], Cohen’s d = 0.74, Hedges’ g = 0.42). Partially created in BioRender. Oppenheim, T. (2026). https://biorender.com/86pe6ky (accessed on 19 June 2026).

A custom benchtop “autobender” apparatus was built and used to mechanically bend each strain sensor to known bend angles while measuring sensor values (Figure 2A). The autobender was made from modular GoBilda hardware (Winfield, KS, USA) and consisted of a stationary plate held parallel to the lab bench, a bending plate that was bent by a SunFounder 20 kg high-torque servo motor between 0 and 90 degrees (Wilmington, DE, USA), a custom PLA 3D printed “knuckle” (radius of 6.35 mm) that bridged the gap between the two plates, and a P3 America Hall effect rotary encoder (ERC 1 0505 S320, 320° measuring range, 0–5 V analog output, 5 V supply, and 6 mm shaft diameter, Leander, TX, USA) that served to report the ground truth bend angles of the plates. An Adafruit BNO055 IMU Breakout Board (New York, NY, USA) was mounted to the bending plate to measure any initial bend angle, typically ~3 deg, and all rotary encoder angles were offset by this amount since the rotary encoder only measured the relative angle.

Strain sensors were attached to the autobender plates using Velcro tabs (Manchester, NH, USA). Tabs were bonded to both sensor ends, and opposing Velcro tabs were bonded to the stationary plate and the bending plate. All tabs were adhered to the sensors or plates with SmoothOn SILPoxy silicone adhesive. Each sensor was positioned with its midpoint centered over the “knuckle” during bending.

To test the sensors, the autobender was bent from approximately 0 deg to 90 deg and back to 0 deg in 15 deg increments while reading data from the rotary encoder and sensor. Five cycles were completed for each sensor. At each 15 deg increment, 100 sensor datapoints were recorded.

### 2.4. Strain Sensor Data Acquisition Circuit

Strain sensor data was amplified and digitized using a voltage divider circuit and op-amp followed by an analog to digital converter (ADC) (autobender, Figure S1A), or a current source and op-amp followed by an ADC (on-hand testing, wireless system, Figures S1B, S12 and S13). See the Supplementary Methods “Strain sensor data acquisition circuit” for more details.

### 2.5. Strain Sensor ADC to Bend Angle Calibration

Based on the correspondence of the sensor values to those of the physics model (Figure 2B), we chose to model the normalized sensor ADC values as a quadratic function of measured bend angle (Figure 2C). In the benchtop testing (autobender) context, we collected a synchronized data stream of ADC values and ground truth bend angles (from the rotary encoder). We fit a quadratic curve between the ground truth bend angle and normalized ADC data $ADC_{i,norm}$. This lightweight calibration approach was selected to prioritize data interpretability, a low computational cost, and ease of deployment on a wearable or paired mobile system.

### 2.6. Sensor Length Testing on the Autobender

Thirteen sensors with identical liquid-metal channel lengths (L_0_) but varying sensor lengths ($L_{1}$) to account for different hand sizes were tested to verify that the sample length does not influence the angular error (Figure 2D). For each sensor, the data was first converted from ADC values to normalized ADC values using Equation (S3) (see Supplementary Methods, “Strain sensor data acquisition circuit”). The synchronized ground truth bend angle data and ADC data were divided into 10 different partitions. For each partition, the remaining 90% of the data was used to fit a calibration curve as described in “Strain sensor ADC to bend angle calibration”, and then used to predict bend angles from ADC values for the left-out 10% of data. This procedure was repeated 10 times, each time with a different 10% of data left out. Bend angle predictions were then pooled across the 10 left-out partitions and compared to ground truth bend angles to assess the absolute angular error for each sample (Figure 2D).

### 2.7. Reapplication Analysis on the Autobender

Three strain sensor samples were evaluated for the consistency of signals after reapplication on the autobender. Each sample was tested during an initial application, then removed from the autobender, reapplied, and tested again. This removal and reapplication procedure was repeated twice per sample, yielding a total of three applications (initial, second, and third) for each sample (Figure 2E).

For each sample and each application, bend angles were predicted using two different calibration curves. One curve was trained on the same sample-application data that was being evaluated (blue boxes, Figure 2F). Notably, the ADC norm values used in this evaluation were calculated using the $ADC_{min},ADC_{max}$ computed from the full sample-application dataset used for fitting and evaluation.

The second calibration curve was trained exclusively on data from the initial application of each sample (Model 1) and then applied, without retraining, to data from the second and third applications of the same sample (orange boxes, Figure 2F). Notably, the ADC norm values used in this evaluation were calculated using the $ADC_{min},ADC_{max}$ computed from the full dataset corresponding to the sample’s first application.

### 2.8. Misalignment Analysis

Three strain sensor samples were evaluated for the consistency of signals after misaligned application on the autobender. Each sample was tested under three mounting conditions: nominal alignment (0 deg), 10 deg misalignment, and 20 deg misalignment (Figure 2G). Misalignment angles were verified using a handheld goniometer prior to data collection.

For each sample and misalignment condition, the absolute angular error was quantified using two types of calibration curves: one trained on the same sample-application data that was being evaluated (blue boxes, Figure 2H), and a second trained on the initial application (aligned, 0 deg) and tested on misaligned data (10 deg and 20 deg misaligned) (orange boxes, Figure 2H). As in the reapplication analysis, the ADC norm values used were calculated using the $ADC_{min},ADC_{max}$ computed from the particular sample-application that was used to fit the calibration curve.

### 2.9. IMU Hardware

The Bosch BNO055 IMU (Reutlingen, Germany), a 9-axis absolute orientation IMU integrating a triaxial accelerometer, gyroscope, and magnetometer, was selected for measuring the orientation of the hand and the forearm. It was chosen for its ease of development enabled by an on-chip sensor fusion algorithm, which can directly output quaternions or Euler angles among other orientation data (including linear acceleration, gravity vector, and calibrated gyroscope rates), freeing up computing resources on the host microcontroller and making deterministic timing in datalogging easier. Its performance in hand kinematics tracking has also been evaluated in the prior literature via benchtop testing to be accurate to less than 4° RMSE in static and dynamic validation cases where the pitch rotation is under 240°/s [15].

In our device, a custom 2-layer PCB was designed to house this sensor in a compact 11.0 mm × 9.5 mm footprint with the goal of mounting the sensor board on the hand in a comfortable and unobtrusive manner to track movement of the hand and forearm (Figure S11). See Supplemental Methods “IMU communication with microcontroller” for further details on how multiple IMUs communicated with the microcontroller housed in the watch case.

### 2.10. Using Two IMUs to Calculate Wrist Flexion/Extension

We estimated the wrist flexion/extension from the IMU placed on the dorsal aspect of the palm (hand-back unit, see below) and the second placed on the dorsal aspect of the forearm inside the watch case. See Supplemental Methods “Wrist flexion/extension calculation details” for details.

### 2.11. On-Hand Strain Sensor Testing

To test the on-hand accuracy and inter-application variability of the strain sensor and the IMU, we conducted a user study. Research was conducted in accordance with the approval of the University of California, Berkeley Institutional Review Board (IRB). All research participants provided informed consent to participate in the study. Berkeley students without any upper limb or hand impairments (*N* = 4) completed the on-hand study (UC Berkeley IRB number: 2023-12-16973). The experimental timeline for the two-hour study consisted of two primary phases. First, participants donned the device to perform a variety of dexterity tasks. Second, to evaluate the system’s robustness to sensor reapplication, the devices were completely removed, reapplied, and the participants performed the same tasks again.

### 2.12. Camera Setup

**Figure 3:**
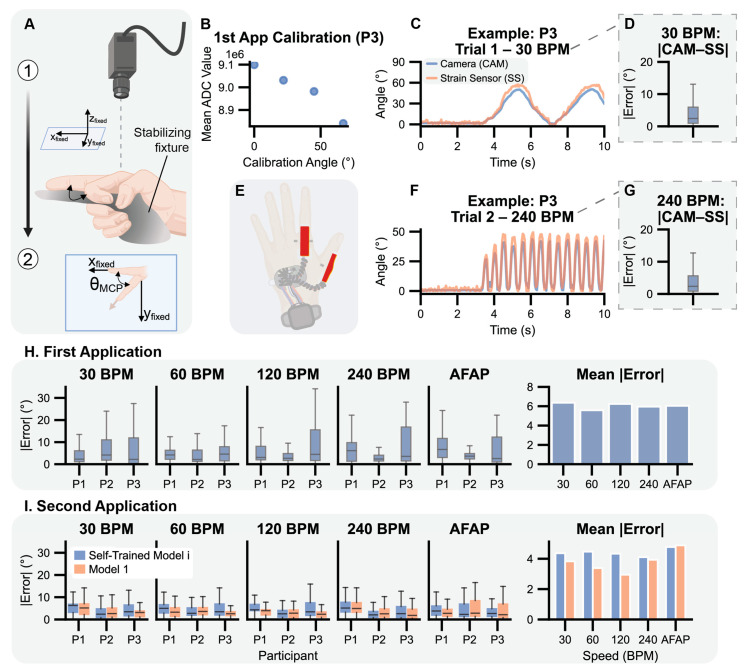
Strain sensor error analysis on the hand. (**A**) Camera and stabilizing fixture for user study to investigate the on-hand sensor accuracy of measuring index MCP flexion/extension. (**B**) Example calibration data from the first sensor application on the third participant (P3). (**C**) Example time-series comparison of the predicted bend angles from strain sensors and the corresponding camera-measured bend angle for the index MCP during the slowest bend rate (30 BPM). (**D**) Absolute angular error box plot computed from the time-series comparison shown in (**C**). (**E**) Schematic showing placement of the strain sensor over the index and thumb MCP joints. (**F**,**G**) Similar to (**C**,**D**), except the time-series data/absolute angular error box plot correspond to the 240 BPM MCP bend rate. (**H**) Summary angular error box plots from the first sensor application for each participant and each MCP bend rate. The bar plot on the far right shows the MAE across all participants for each MCP bend rate. (**I**) Second-application summary. Blue boxes represent errors derived from a calibration curve fit using second-application data (“self-trained model”), while orange boxes represent errors obtained by applying the first-application calibration to the second-application data (“Model 1”). When MAEs were averaged across speeds within each participant, no statistically significant difference was detected between the two calibration approaches (paired *t*-test, t(2) = 1.20, *p* = 0.35). The estimated mean difference was −0.61° (95% CI [−2.79°, 1.57°], Cohen’s d = −0.70, Hedges’ g = −0.40). A linear mixed-effects model with speed as a fixed effect and participant as a random effect on intercept, pooled across participants and applications, revealed no significant effect of speed on the MAE (slope = 0.096, CI [−0.252, 0.443], z = 0.539, N = 45, *p* = 0.59, R^2^ marginal = 0.007, R^2^ conditional = 0.007). Partially created in BioRender. Schlegel, H. (2026). https://biorender.com/t2ywslt (accessed on 19 June 2026).

**Figure 4:**
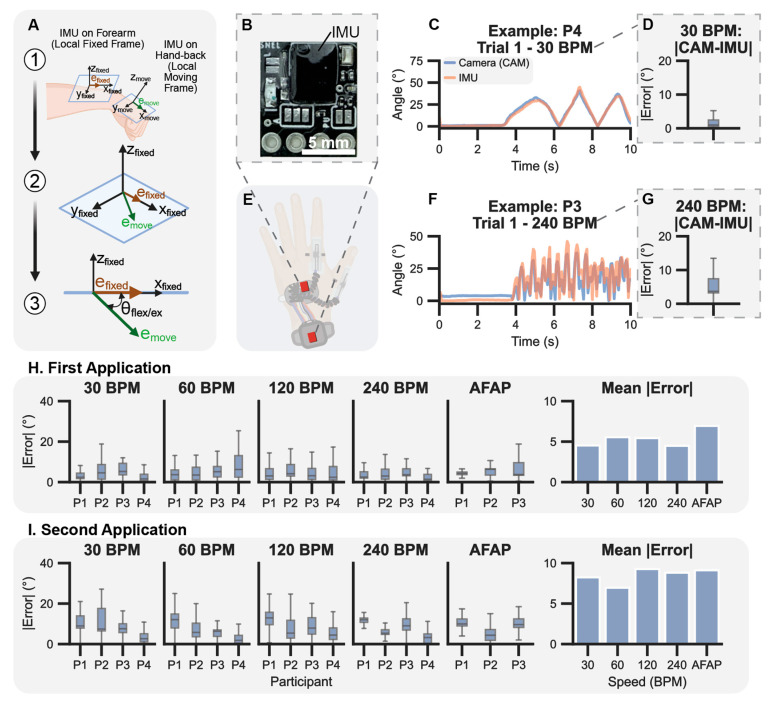
IMU error analysis on the hand. (**A**) Schematic illustrating the plane-method calculation used to measure the palm flexion angle from two IMUs. (**B**) Photograph of the customized breakout board designed for the BNO055 IMU. (**C**) Example time-series comparison of predicted bend angles from the IMU (orange) and the corresponding camera-measured bend angle (blue) for wrist flexion/extension during the slowest bend rate (30 BPM). (**D**) Absolute angular error box plot computed from the time-series comparison shown in (**C**). (**E**) Schematic showing IMU placement within a watch case and on the dorsal surface of the palm, with both IMUs oriented in the same direction. (**F**,**G**) Similar to (**C**,**D**), except the time-series data/absolute angular error box plot correspond to the 240 BPM bend rate. (**H**) Summary angular error box plots from the first application for each participant and each wrist bend rate. The bar plot on the far right shows the mean angular error across all participants for each wrist bend rate. (**I**) Same as (**H**) but for the second application. After averaging the MAE across the first and second applications within each participant and within each movement speed, a linear mixed-effects model with speed as a fixed effect and participant as a random intercept revealed a significant effect of speed on the mean IMU MAE (slope = 0.585 deg per speed category, z = 2.018, N = 19, *p* = 0.04356, 95% CI [0.017, 1.152], R^2^ marginal = 0.114, R^2^ conditional = 0.508). Partially created in BioRender. Schlegel, H. (2026). https://biorender.com/cxnk361 (accessed on 19 June 2026).

To test the on-hand accuracy of the strain sensor and IMU, participants wore the strain sensor on their index MCP and wore the IMUs on the dorsal aspect of their hand and inside the smart watch. As illustrated in Figure 3A and Figure 4A, an overhead camera (Teledyne FLIR Blackfly S USB3 1.6-MP color camera (CS mount), Wilsonville, OR, USA) equipped with a Tamron M118FM08 megapixel fixed-focal industrial lens (8 mm) (Saitama, Japan), with data acquisition handled by a Raspberry Pi (Cambridge, UK) via a Hirose HR10 circular connector (HR10A-7P-6S, Downers Grove, IL, USA), was set up to serve as a ground truth measurement for joint angles.

The camera was mounted 66.5 cm from a lab bench to image a field of view that encompassed the intended (width × length) movement area. Bubble levels were affixed to the sides of the camera’s chassis along two orthogonal axes in a plane perpendicular to the benchtop to ensure the camera’s optical axis was vertical.

### 2.13. Testing Protocol

Using a BIC BodyMark Temporary Tattoo Marker (Shelton, CT, USA), anatomical landmarks were marked on the radial aspect of the hand and forearm (Figure S2). Marks were placed at (1) the index finger proximal interphalangeal (PIP) joint, (2) the index finger metacarpophalangeal (MCP) joint, (3) the midpoint along the index finger metacarpal between the MCP joint and the wrist, (4) the distal aspect of the radial wrist near the wrist joint, selected to minimize skin stretch during wrist flexion, and (5) a second point located approximately 2 inches proximal to the wrist along the same radial forearm axis. With the hand positioned on its side along a custom fixture that helps keep the index finger aligned with the palm (Figure 3A, “stabilizing fixture”), the marked landmarks were aligned using a ruler to ensure that all segments were initially collinear, thereby minimizing initial bend angle offsets and approximating a starting joint angle of 0°.

After skin marker landmarks were placed, the strain sensor, IMUs, and associated digitization and communication electronics were applied to the participant’s right hand following the donning/doffing protocol (see “Application of the watch, HBU, and IMU” and “Strain sensor donning and doffing”” in the Supplementary Methods). To calibrate the sensors on-hand, participants placed their hand on the aforementioned custom fixture consisting of an ergonomic handle with a flat side wall and a short, fan-shaped platform primarily designed to constrain the index finger to a plane perpendicular to the camera view. This fixture helps keep the palm stationary and perpendicular to the bench top and has a marking that facilitates index finger–palm alignment (0°). Static recordings of ~10 s were collected with the index finger placed and held at the following angles between markers (2), (3), and (4) measured by a protractor: 0°, 22.5°, 45°, and 67.5°. After calibration, four tasks were performed: (1) index finger flexion at various speeds; (2) wrist flexion at various speeds; (3) cylinder reach and grasp (not described, data not included); and (4) small cube handling (not described, data not included).

#### 2.13.1. Index Finger Flexion

Participants performed repeated index MCP flexion–extension cycles (0° to 90° or maximum flexion) at five speeds: slow (4 s/cycle; metronome at 30 bpm), medium (2 s/cycle; 60 bpm), fast (1 s/cycle; 120 bpm), very fast (0.5 s/cycles; 240 bpm), and as fast as possible (without metronome). Participants were instructed to keep their index fingers aligned with the index finger–palm alignment marker (0°) before the start of every trial, and to keep their index fingers flat on the fan-shaped platform perpendicular to the camera throughout each trial. Two repetitions were collected at each speed. For every trial, 10 s of footage was recorded, and signals of “Ready,” “Set,” and “Go” were automatically displayed in 1 s intervals on a screen shown to the participant to instruct the onset of movement, to increase the trial-to-trial timing consistency and to collect baseline data before the onset of movement. The TonalEnergy metronome was used on a smartphone with an animated inverted pendulum visualization (akin to mechanical metronomes), and participants were instructed to synchronize with the pace and direction of the pendulum and change their flexion–extension direction at every audible click, with minimal dwell time when changing directions. The metronome was started by the experimenter and was synchronized with the “Go” cue.

#### 2.13.2. Index Finger Abduction

To test how sensitive strain sensors were to movements about the MCP joint that were not MCP bending, we monitored strain sensor signals while participants completed isolated abduction movements of the index finger that were verified with captured camera videos. Participants placed their open hands palm-down on the table, overlaid on a protractor, and moved their index finger back and forth to approximately 20° (average maximum index finger abduction possible [25]) for 10 s. All other fingers remained stationary.

#### 2.13.3. Wrist Flexion

Like the index finger flexion task, participants performed repeated wrist flexion–extension cycles at the same five speeds: slow (4 s/cycle; metronome at 30 bpm), medium (2 s/cycle; 60 bpm), fast (1 s/cycle; 120 bpm), very fast (0.5 s/cycle; 240 bpm), and as fast as possible (without metronome). Participants were instructed to hold the hand fixture with an extended index finger and keep the same hand posture while they flexed and extended their wrists, so that their palms were kept perpendicular to the bench top surface during movement. Participants started movement with their wrists at 0° (measured between markers (3), (4), and (5)), and were instructed to flex their wrists to the full range of motion before extending to the full range of motion and repeating. Start time priming and metronome pacing cues were similarly used. Two other tasks (the cylinder reach and grasp task, and cube manipulation task) were performed but data from these tasks is not reported here.

The pose-tracking software DeepLabCut (DLC) version v3.0.0rc10 [26] was used to extract the marked points on the skin from camera images. To measure the ground truth MCP bend angle, the angle between the vectors defined by points 2–1 and 2–3 were calculated. To measure the ground truth of the wrist flexion/extension bend angle, the angle between the vectors defined by points 4–3 and 4–5 were calculated. Tracked points with likelihood scores of <0.45 were removed to avoid poor camera tracking (often due to blurred images or blurred markers) from influencing results. Note that data from participant 4 (P4) was excluded in the “as fast as possible” wrist flexion condition due to their camera tracking having >50% of datapoints rejected due to low likelihood scores (Figure 4H,I).

### 2.14. On-Hand Strain Sensor Testing Data Analysis

We evaluated the on-hand strain sensor accuracy using a calibration curve that was formed (1) by using calibration data from the same application as used for testing, and (2) by using calibration data from the first application to assess accuracy on the second application. In both cases, calibration curves were fit by having the participant bend their index finger MCP angle to a known set of angles while collecting sensor data (as measured by a finger goniometer): 0, 22.5, 45, and 67.5 degrees. Sensor data from each calibration angle were concatenated and the overall dataset was fit with a quadratic curve (Figure 3B).

The accuracy of the predicted angles from using the self-train calibration curve is displayed in the blue bars in Figure 3H,I, and the accuracy of the predicted angles from using the first application calibration curve is displayed in orange bars in Figure 3I. To account for differences in sensor mounting and baseline strain between applications, the second-application data were first normalized to the range [0, 1] based on their own $ADC_{min}$ and $ADC_{max}$ (Equation (S3)) observed during the second-application calibration (corresponding to 67.5° and 0° flexion, respectively).

This rescaling step was necessary because different sensor applications did not produce identical minimum and maximum ADC values at 67.5° and 0° flexion. Without rescaling, directly applying the first-application calibration curve to second-application data scaled according to $ADC_{min}$ and $ADC_{max}$ from the first application resulted in physiologically implausible bend angles exceeding 90°, substantially increasing the absolute angular error. Notably, this procedure differed from the Model 1 procedure performed on the autobender, which is why Model 1 errors were higher on the autobender than self-trained model errors (Figure 2F), but not on-hand (Figure 3I). 

### 2.15. Wireless System Description

The wireless wearable was designed to mount sensors and electronics comfortably for multiple hours of use. The main components are IMU sensors, “e-skin” strain sensors, the hand-back unit, the strain sensor signal acquisition board, the microcontroller, and the battery, which were secured on the back of the hand with adhesive or mounted in a watch enclosure. A high-level electronics overview is shown in Figure S13.

The watch enclosure housed the strain sensor acquisition circuit, microcontroller, battery, and one of the two IMU sensors in the system that tracks wrist movement. The watch case was designed to slide onto an off-the-shelf silicone-encased bistable spring band (a “slap band” that coils to wrap around the wrist when tapped). The watch case and the watch strap can be applied using one hand holding the slap band with a simple power grip (unlike typical watch straps which require considerable dexterity).

**Figure 5:**
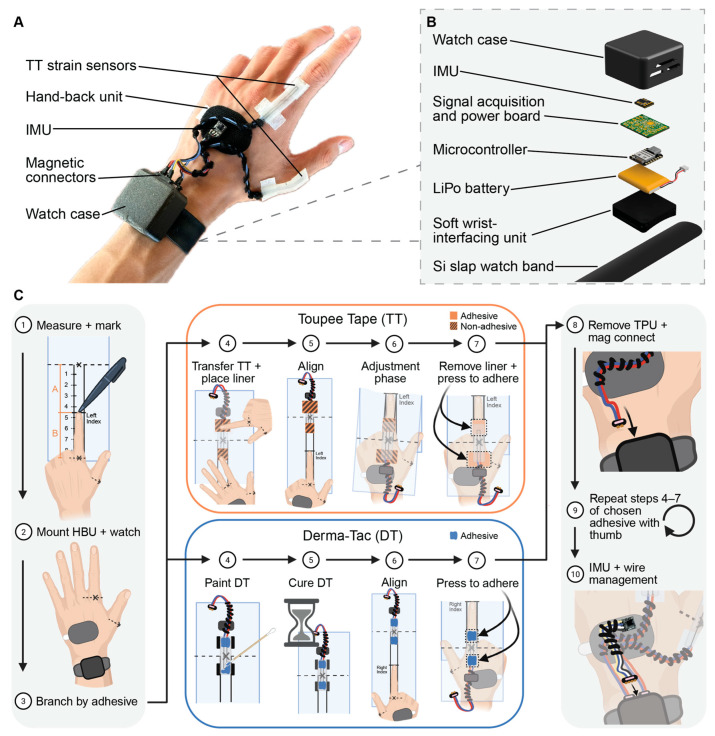
The full wireless kinematics system. (**A**) Full wireless kinematics system on the hand. (**B**) Exploded view of watch case. (**C**) Donning/doffing strategy for the on-hand kinematics system. Participants first measure and mark joint centers (X), and mount the watch case (mag connectors outward) and hand-back unit (HBU). The application flow then branches by adhesive: Toupee tape (TT) in orange uses a dry-alignment step that allows adjustment before adhesion, followed by pressing over TT pad zones and connecting the magnetic lead. Derma-Tac (DT) in blue is painted onto the pad guides (maintaining a consistent adhered surface area with the TT methodology), cured for at least 2 min, and then aligned and adhered in a single, non-adjustable press. Both application processes are the same for the final steps: re-press the pads to secure the sensors, attach the IMU, and settle the wires. Partially created in BioRender. Schlegel, H. (2026). https://biorender.com/kg390w6 (accessed on 19 June 2026).

The hand-back unit (HBU) consisted of a 5 × 3 cm area of two layers of materials: the top layer was hook-side Velcro strips (QTBLY) and the bottom layer that attached to the hand was a double-sided medical adhesive tape (toupee tape, Vapon, Fairfield, NJ, USA). The HBU was attached to the dorsal aspect of the palm (see Figure 5A) and served as an anchor site for the IMU and IMU PCB that were placed on the back of the hand, as well as for the strain sensor wires.

More complete descriptions of the different components, as well as the firmware and web application that acquired data, can be found in Supplemental Methods “Detailed wireless system description”.

### 2.16. Application Procedure for Wireless System

The full wireless system consisted of (i) a watch-style electronics housing worn at the wrist with magnetic connectors oriented distally (towards the hand), (ii) a dorsal hand-back unit (HBU) for wire management, (iii) two strain sensors (index MCP and thumb MCP) mounted on reusable thermoplastic polyurethane (TPU) sensor applicators, and (iv) an inertial measurement unit (IMU) mounted on the HBU (Figure 5A,B). The watch and HBU were donned first (Figure 5C, step 2), then the two strain sensors were applied (index followed by thumb; Figure 5C, steps 4–9), the strain sensor magnetic leads were connected to the watch, the IMU was attached to the HBU (Figure 5C, step 10), and the wires were organized on the HBU (Figure 5C, step 10), and finally the strain sensors were pressed at the adhesive contact regions to ensure full adhesive contact. See Supplemental Methods “Application of the watch, HBU, and IMU” for further details.

There were two strain sensor application techniques tested: toupee tape (TT) and Derma-Tac (DT). The TT method used strain sensors that were fabricated with mounting squares. Double-sided toupee tape was then applied to the mounting squares, and the strain sensor was applied to the hand. The DT method used a commercial liquid silicone-to-skin adhesive (Derma-Tac) that was applied directly to select locations on the sensor and allowed to dry for at least 2 min prior to application to the hand. Both methods used a measurement tool and alignment tool to ensure sensors were correctly placed over the MCP joint. See Supplemental Methods “Strain sensor donning and doffing” and Figures S5–S8 for further details.

## 3. User Comfort Study Protocol

### 3.1. Participants

Participant recruitment and all procedures were approved by the University of California, Berkeley Institutional Review Board (UC Berkeley IRB number: 2023-12-16973). Written informed consent was obtained from all participants. We recruited n = 4 adults with typical healthy upper-limb movement (four female; age 21.25 ± 1.64, range 20–24; right-handed).

### 3.2. Study Design

**Figure 6:**
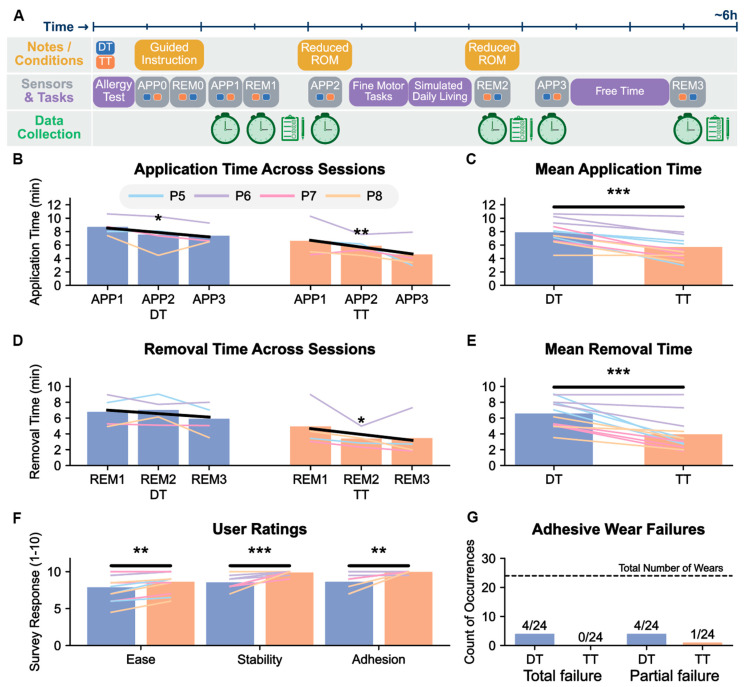
User comfort study protocol and comparative performance of two sensor-attachment methods (DT, blue vs. TT, orange). (**A**) Study timeline. Participants completed an allergy check, experimenter-guided APP0/REM0, then three timed cycles of application and removal without any experimenter feedback (APP1–3, REM1–3), indicated by the green clock icon. Fine motor tasks (pinch/grasp, writing, and typing) and daily-living tasks (eating, phone use, hand sanitizer application, and jacket on/off) followed APP2; APP2/REM2 were performed with finger splints to limit finger range of motion. A ~60 min free-time block preceded REM3. Cycle-specific questions followed each APP/REM cycle (indicated by the clipboard icon); final open-ended questions were asked prior to concluding the session. (**B**) Application time by method and cycle with participant traces in gray and mixed-effects learning lines in black. DT and TT both get significantly faster with each application (linear mixed-effects model, random intercept by participant, DT: slope = −39.5 s per application, 95% CI [−74.2, −4.78], t = −2.23, *p* = 0.026, R^2^ marginal = 0.092, R^2^ conditional = 0.796, n = 12, TT: slope = −60.8 s per application, 95% CI [−99.5, −22.0], t = −3.07, *p* = 0.0021, R^2^ marginal = 0.143, R^2^ conditional = 0.833, n = 12). (**C**) Pooled over APP1-3, TT applications were significantly faster than DT applications (difference between mean application times with DT and TT was 130.6 s, 95% CI [−180.06, −81.11], paired, two-sided *t*-test, t = −5.810, *p* = 0.0001, n = 12 paired observations from 4 participants × 3 APP, Cohen’s d = −1.677, Hedges’ g = −1.560). (**D**) Removal time by method and cycle with participant traces in gray and mixed-effects learning lines in black. DT did not significantly change in removal time but TT significantly improved in speed (linear mixed-effects model, random intercept by participant, DT: 95% CI on slope [−60.48, 7.98], t = −1.503, *p* = 0.133, R^2^ marginal = 0.036, R^2^ conditional = 0.824, n = 12, TT slope: −44.4 s per removal, 95% CI [−87.23, −1.52], t = −2.030, *p* = 0.0424, R^2^ marginal = 0.070, R^2^ conditional = 0.812, n = 12). (**E**) Pooled over REM1–3, TT removals were significantly faster than DT removals (difference between mean removal times between DT and TT of 157.9 s, 95% CI [−226.60, −89.23], paired, two-sided *t*-test, t = −5.060, *p* = 0.0004; n = 12 paired observations = 4 participants × 3 REM, Cohen’s d = −1.461, Hedges’ g = −1.359). (**F**) Survey ratings (1–10; paired, two-sided *t*-tests; n = 12 pairs/metric) favor TT on ease (+0.75, 95% CI [0.33, 1.17], t = 3.954, *p* = 0.0023, Cohen’s d = 1.141, Hedges’ g = 1.062), stability (+1.33, 95% CI [0.79, 1.88], t = 5.360, *p* = 0.00023, Cohen’s d = 1.547, Hedges’ g = 1.439), and adhesion (+1.33, 95% CI [0.60, 2.07], t = 4.000, *p* = 0.0021, Cohen’s d = 1.155, Hedges’ g = 1.074). (**G**) Wear failure summary (counts across WEAR1–3): total failure (fully off) and partial failure (lifting) for DT and TT; *y*-axis shows counts (0–24). The 24 count arises from 4 participants × 3 APP × 2 sensors/application. For full figure, * indicates *p* < 0.05, ** indicates *p* < 0.01, *** indicates *p* < 0.001. Partially created in BioRender. Schlegel, H. (2026). https://biorender.com/cndws0y and https://biorender.com/xn0edbu (accessed on 19 June 2026).

Participants completed a single ~6 h session comparing DT and TT attachment methods in a within-subject design (Figure 6A). To reduce participant confusion, DT-mounted sensors were assigned to the right hand and TT-mounted sensors to the left hand for all participants (left hand guided DT application onto right hand; right hand guided TT application onto left hand). Two sensors (thumb MCP and index MCP) and the full wearable system (watch, HBU, and IMU) were used for each method.

### 3.3. Session Timeline

Participants first completed a 30 min allergy/skin-tolerance check. A small amount of DT was applied to the dorsal hand skin, and a single TT piece was placed on the dorsal hand skin. The experimenter monitored for irritation (e.g., redness, itching, or discomfort) and participants self-reported any sensations during the 30 min observation period. Participants also self-reported any sensations or discomfort during the entire session.

Next, participants completed a fully guided application and removal (APP0/REM0) process for both DT and TT attachment methods, during which the experimenter walked them through each step and provided tips and coaching. Participants then completed three timed, self-application and removal cycles (APP1–APP3 and REM1–REM3) for each method without any tips or coaching from the experimenter. The intervals between each application and the subsequent removal were defined as wear cycles (WEAR1–WEAR3). Each complete cycle (APP, WEAR, and REM) was followed by verbally administered cycle-specific questions (Supplementary File S1).

To assess usability under reduced dexterity, APP2 and REM2 were performed while participants wore rigid oval finger splints (UAKIKAU) that limited finger range of motion. During WEAR2, participants completed fine-motor tasks (pinch/grasp, writing, and typing) and simulated activities of daily living (eating, phone use, sanitizer/wet exposure, and clothing on/off). During WEAR3, participants completed a ~60 min free-time wear block prior to REM3. The session concluded with participants completing the remainder of the user questionnaire (Supplementary File S1: Sections 2–4).

### 3.4. Outcome Measures and Recording

The primary usability outcomes were the application time and removal time for each method and cycle, recorded using start/stop timestamps on a standardized administration sheet (Supplementary File S2). The application time was recorded from the participant’s first interaction with the wearable components at the start of the application sequence (watch/HBU donning) to the completion of application, defined as both sensors adhered to the skin at their adhesive contact regions and magnetically connected to the watch module (Figure 5C). The removal time was timed from the participant’s first interaction with the system for removal (initial disconnect/peel actions) to the completion of removal, defined as both sensors removed and secured back onto their TPU applicators and the watch/HBU removed.

Secondary outcomes included: (i) cycle-specific questions completed after each full cycle (application, wear, and removal) consisting of Likert-scale (1–10) ratings in domains including the ease of application, freedom of movement, stability during motion, adhesion over time, wiring management, and overall experience (full items in Supplementary File S1, Section 2), (ii) a final round of questions with open-ended feedback aimed at evaluating the user experience, sensor failures, and adhesive method preferences (Supplementary File S1, Sections 3 and 4); and (iii) observed wear failures logged by the experimenter (Supplementary File S2) and categorized as total failure (sensor fully detached) or partial failure (lifting/edge peel), counted across wear cycles. The experimenter additionally logged the sensor IDs, notes/photos, and a basic sensor connectivity indicator (watch LED status) at key steps (Supplementary File S2).

### 3.5. Long-Term Wear Protocol

**Figure 7:**
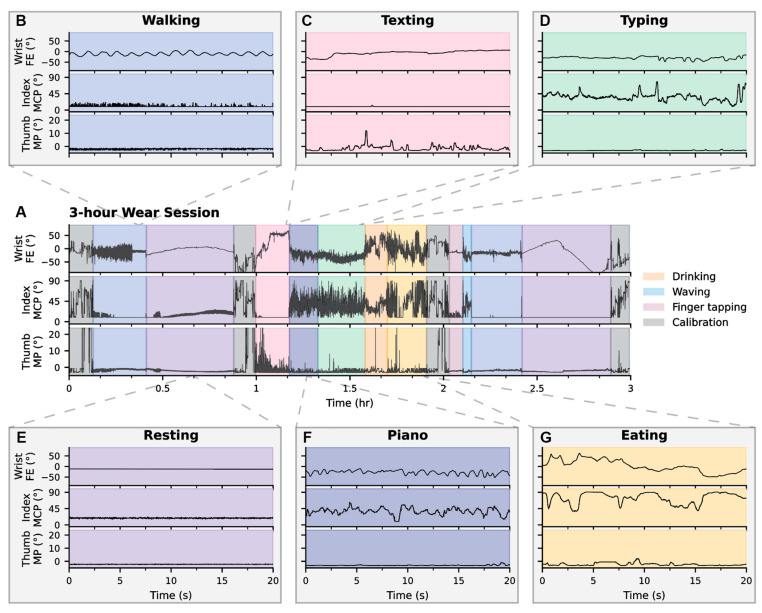
Long-wear recording of the hand kinematics system during daily activities. (**A**) Representative segment of the wear session, with shaded regions indicating labeled activity periods and calibration blocks. (**B**–**G**) Representative 20 s windows are shown for walking, texting, typing, resting, piano, and eating, with the wrist flexion/extension (top), index MCP bend angle (middle), and thumb MP bend angle (bottom). (**B**) During walking, nothing was held in the hand. (**C**) During texting, the phone was held between the index finger and thumb while the wrist and arm remained relatively stationary. (**D**) Typing was performed using a low-profile keyboard. (**E**) During resting, the hand remained stationary on the desk. (**F**) The piano segment reflects a beginner pianist, and (**G**) the eating segment includes a mixture of typical eating-related movements. For this participant, the plotted *y*-axis ranges were selected to reflect a comfortable range of motion during natural activities: approximately −60 to +70° for wrist flexion/extension, 0 to 90° for index MCP, and 0 to 20° for thumb MP. Index MCP and thumb MP bend angles were estimated from sensor ADC values using the first calibration block collected immediately after device application.

To evaluate the system performance during extended, naturalistic use, a participant wore the full wireless hand kinematics system continuously for approximately 9 h while performing activities of daily living (Figure 7). These activities included walking, texting, typing, resting, piano playing, and eating, among others. A representative 3 h segment of the session is shown in Figure 7A, with example 20 s windows of specific activities shown in Figure 7B–G.

At multiple timepoints throughout the session, intermediate calibration checkpoints were performed to assess the stability of the strain sensors over time. Calibrations were performed at the time of initial wear (0 h), 1 h after application, 2 h after application, and 4 h after application (Figure S9). During each calibration block, the participant positioned their index finger MCP joint at a set of target angles (0, 22.5, 45, 67.5, and 90°), referenced using a handheld finger goniometer. Each target angle was held for approximately 10 s while the sensor data were recorded. This procedure was repeated for the thumb MP joint. Each calibration took a total of about 7 min.

Sensor data from each calibration block was used to generate normalized calibration curves for the index and thumb (Supplementary Figure S9B,C).

### 3.6. Statistics

Statistical analyses were performed in Python 3.12.3 using the NumPy (version 1.26.4), SciPy (version 1.13.1), Pandas (version 2.2.2), and Statsmodels (version 0.14.2) packages. Tests included an ANOVA, paired *t*-tests, and linear mixed-effect models. Statistical significance was assessed using a significance level of (alpha = 0.05). However, traditional parametric null-hypothesis significance testing carries the risk of experimental underpowering. To ensure statistical validity, we supplemented our parametric comparisons with standardized effect sizes and uncertainty estimates. In addition to *p*-values, effect sizes and 95% confidence intervals were reported. Effect size reporting varied depending on the statistical test. For paired *t*-tests, Cohen’s d values and Hedges’ g values (correction to Cohen’s d values for the small sample size) were reported. For ANOVA tests, $\omega^{2}$ values were reported [27]. For linear mixed-effects models, the variance explained was quantified using Nakagawa’s marginal and conditional $R^{2}$, representing the variance explained by fixed effects alone and by fixed plus random effects, respectively. We note that restricted engineering and electronic device testing paradigms face a similar challenge with underpowered statistics, and deal with this via constrained Taguchi–ANOVA optimizations that also focus on effect-size results [28]. 

## 4. Results

As a starting point for our wearable system, we sought to develop thin, flexible, unobtrusive strain sensors that could measure finger bend angles. We developed thin, flexible silicone “e-skin” liquid metal bend sensors that were fabricated in the lab according to the protocol outlined in Figure 1, inspired from prior work in flexible, wearable sensors [29,30,31,32,33,34,35]. Sensors were ~43 mm sensor length ($L_{0}$) × 11 mm sensor width × 3 mm sensor thickness (Figure 1B), though some were fabricated to be shorter/longer in length to accommodate shorter/longer fingers. This fabrication process could be easily adapted to make sensors of different lengths or widths for different finger joints.

### 4.1. Benchtop Testing of Strain Sensors

We sought to test the accuracy of our strain sensors on the benchtop. Sensors were tested on a custom-made “autobender” (Figure 2A). According to the theoretical physics model (Equation (1)) of how the resistance of the microfluid channel should change with bending, there should be a quadratic relationship between the axial strain ($\epsilon_{XX}$) and change in resistance ($\Delta R/R_{0})$. This relationship should change as a function of the cross-sectional area (CXA) of the channel. Figure 2B shows the $\Delta R/R_{0}$ data collected from three sensors with various strains applied and the theoretical model predictions of the $\Delta R/R_{0}$ of these sensors as a function of strain (dotted lines). The R^2^ (variance accounted for) between the data and model was 0.999, 0.994, and 0.993 for the three samples. We find that when normalized sensor values (voltages) are plotted against the measured bend angle (instead of $\epsilon_{XX}$), the quadratic trend is retained (Figure 2C). This approach of plotting the known bend angle versus the normalized sensor voltage is used to create a “calibration curve” for each sensor.

We next measured how well each sensors’ calibration curve predicted the bend angle on the held-out test bending data. We specifically wanted to test whether shorter or longer sensor lengths ($L_{0}$) would be associated with different magnitude errors. We tested 13 samples of varying lengths on the autobender and built calibration curves using a “training” set of data and predicted bend angles on a held-out “test” set of data from each application. The distribution of absolute differences between the ground truth and predicted bend angles is shown in Figure 2D. The mean absolute error (MAE), averaged across samples, was 1.39 deg, with a corresponding sample root mean squared error (RMSE) of 1.75 deg. Additionally, 99.7% of predictions exhibited absolute angular errors ≤ 5 deg. A one-way ANOVA performed on per-sample mean absolute errors showed no statistically significant effect of the sensor length ($L_{0}$) on the absolute angular error (Figure 2D, F(4, 8) = 1.41, *p* = 0.31, unbiased population effect size $\omega^{2}$ = 0.11, a moderate-to-large effect). Because only 13 sensor samples were available across five length categories, and because the estimated effect size is moderate-to-large, sensor length may account for a modest proportion of the observed variability in error, requiring larger studies to more certainly resolve the effect.

We next sought to characterize how well a calibration curve computed from the initial application of the sensor to the autobender generalized to reapplications. In theory, if we could derive a calibration curve for each sensor that was very stable across applications then we could manufacture sensors along with a calibration curve and there would be no need for individual users to have to perform calibration of the sensor again. We applied a sensor to the autobender, collected data, and created a calibration curve (“Model 1”). We then reapplied the sensor to the autobender and tested whether predicted angles using Model 1 (orange boxplots) were significantly worse than angles predicted using a model that was trained on separate training data from that same application (“self-trained model”, blue boxplots) (Figure 2E,F). We found that when MAEs were computed within each sample across reapplications, Model 1 produced significantly higher errors than the corresponding self-trained models (paired *t*-test, t(2) = 6.72, *p* = 0.02). The mean paired increase in MAE was 0.64° (95% CI [0.23°, 1.06°]), corresponding to a large effect size (Cohen’s d = 3.83, Hedges’ g = 2.19). Although the use of Model 1 after reapplication was significantly worse than use of Model 1 in the initial application, errors overall were very low: when predictions were pooled across samples and applications, the MAE of Model 1 estimates was 1.79 deg, with a corresponding RMSE of 2.11 deg. Additionally, 99.21% of Model 1 predictions exhibited absolute angular errors ≤ 5 deg. To assess whether the performance continued to change with additional reapplications, MAEs from the second and third reapplications were compared using Model 1. No statistically significant difference was detected between the second and third reapplications (paired *t*-test, t(2) = −0.82, *p* = 0.50). The estimated mean difference was −0.43° (95% CI [−2.66°, 1.81°], Cohen’s d = −0.47, Hedges’ g = −0.27). We conclude that there is no evidence for a systematic change in performance between the second and third reapplications.

Finally, we sought to test how critical it was that sensors were applied in a way that is perfectly aligned to the bending axis of the autobender. If the calibration curves were not sensitive to slight misalignments, this would allow for a less accurate sensor-to-hand application strategy. Using an approach like our reapplication testing, we first applied the sensors to the autobender at 0 degrees of misalignment and trained a calibration curve, “Model 1”. We then tested this model on held-out data from this same application (split blue/orange box plots in Figure 2H). We then removed and reapplied the sample at a 10 degree misalignment and 20 degree misalignment (Figure 2G) and report the absolute angular errors of predictions either from “Model 1” (orange box plots in Figure 2H) or from “self-trained” models (blue box plots in Figure 2H). No statistically significant difference was detected between the two calibration approaches (paired *t*-test, t(2) = 1.29, *p* = 0.33). The estimated mean difference in the MAE was 1.67° (95% CI [−3.79°, 7.13°]), corresponding to a moderate effect size (Cohen’s d = 0.74, Hedges’ g = 0.42). We conclude that there is no evidence for a systematic change between the self-trained models and Model 1 under sensor misalignment conditions. When predictions were pooled across samples and applications, the MAE of Model 1 estimates was 2.31 deg, with a corresponding mean per-sample RMSE of 2.80 deg. Additionally, 91.3% of Model 1 predictions exhibited absolute angular errors ≤ 5 deg.

### 4.2. On-Hand Testing of Strain Sensors

Having established that the strain sensors were accurate on the benchtop, and our lack of evidence that they changed in accuracy with sensor length, reapplication, and misalignment, we next tested the on-hand accuracy of the strain sensors (Figure 3). We specifically tested the accuracy of the sensor on the index finger; the on-hand accuracy of the thumb remains uncharacterized. Participants (N = 4) were recruited and had strain sensors applied to their index finger using the toupee tape attachment method (see Section 2.16). Our general approach was to track ground truth index finger MCP bending with a high-speed camera while collecting synchronized strain sensor data. To do this, we developed a stabilizing fixture that allowed users to bend their index finger in a plane parallel to the table they were resting on and parallel to the image plane of an overhead camera (Figure 3A). We developed a calibration curve for each user and sensor by asking users to bend their fingers to specific angles (0, 22.5, 45, and 67.5 degrees) while collecting strain sensor data and validating the bend angle with a finger goniometer (Figure 3B). We then asked users to bend their index finger back and forth at different speeds that were cued using a metronome. Absolute angular errors were computed between sensor-predicted bend angles and camera-measured bend angles (Figure 3C,D,F,G). One out of the four participants (P4) had an issue with sensor adhesion in their first application (Figure S3A), making their angular errors extremely high. However, in their second application, adhesion was perfect and their angular errors were comparable with the other participants (Figure S3B).

Excluding P4, on-hand measurements produced higher absolute angular errors than benchtop testing. For the first application, the MAE averaged across participants and bend rates was 6.03 deg, with a corresponding mean RMSE of 9.01 deg. For the second application, the MAE was 4.41 deg (mean RMSE = 6.51 deg) when a new calibration curve was derived from second-application data (self-trained model). When predictions were generated using the calibration curve from the first application (Model 1), the MAE was 3.80 deg (mean RMSE = 5.58 deg). No statistically significant difference was detected between the two calibration curve approaches for the second application (paired *t*-test, t(2) = 1.20, *p* = 0.35). The estimated mean difference was −0.61° (95% CI [−2.79°, 1.57°]), corresponding to a moderate effect size (Cohen’s d = −0.70, Hedges’ g = −0.40). The data do not provide strong evidence favoring either calibration approach (Figure 3I). Averaging across the first-application self-trained condition and the second-application Model 1 condition yielded an overall MAE of 4.91 deg, with a mean RMSE of 7.30 deg across the two application conditions. When MAEs from these two conditions were pooled across participants, speeds, and applications, 88% were ≤10 deg.

To assess the influence of the bend speed on the prediction accuracy, a linear mixed-effects model was fit with speed as a fixed effect and participant as a random intercept. No statistically significant association between the bend speed and MAE was detected (slope = 0.096, 95% CI [−0.252, 0.443], z = 0.539, *p* = 0.59, R^2^ marginal = 0.007, R^2^ conditional = 0.007). These results do not provide evidence of an effect of speed on sensor accuracy.

#### Comparison of Calibration Procedures for Autobender and On-Hand Testing

The autobender and on-hand studies both evaluated reapplication of the sensor and reuse of a previously derived calibration model (“Model 1”) in Figure 2F,H and Figure 3I, respectively. We found that in the on-hand study, an additional calibration step was needed to keep errors low when reusing “Model 1”. In the autobender results in Figure 2F,H, “Model 1” predictions were done after using the first application’s ADC minimum and maximum to normalize the second application data (“autobender calibration method”). In the on-hand study, data from the second application was normalized according to its own minimum and maximum range and then the first application calibration curve was applied (“on-hand calibration method”).

If we used the “on-hand calibration method” for the on-hand study, we found that using Model 1 produced an average MAE of 3.80° and an average RMSE of 5.58°, as reported above. When using the “autobender calibration method”, using Model 1 produced an average MAE of 9.11° and an average RMSE of 12.17°, corresponding to increases of approximately 140% and 103%, respectively.

When sensors are reapplied to the autobender, the autobender is consistently at a zero degree angle (flat, not bent), and there are lines indicating where the sensor edges should be placed. Consequently, both the baseline ADC value and the dynamic ADC range remained relatively stable from application to application. Model 1 was therefore applied using the original calibration relationship and the original normalization range derived from the first dataset.

When the sensors are reapplied to the hand, it is possible the finger is slightly more or less bent at its initial posture. We also suspect there is also more variability in the sensor placement on the hand since it involves multiple transfer processes (toupee tape transfer to hand, sensor placement on TPU, and sensor transfer from TPU to hand) whereas the autobender just has one transfer (transfer sensor to autobender). Finally, depending on how well the sensor pads line up with the toupee tape, it is possible that there is variability in the strain transfer between the finger and the sensing element. Altogether, we hypothesize that these factors can result in distinct baseline ADC values and dynamic ADC ranges from application to application in the on-hand testing. Thus, normalization and endpoint rescaling (i.e., the “on-hand calibration method”) serve an important role in compensating for application-dependent shifts in signal offset and gain while preserving the calibration relationship derived from the first application.

### 4.3. Strain Sensor Sensitivity to Off-Axis Movement

We additionally tested how sensitive the strain sensors were to movements that were not pure bending of the MCP joints. Participants completed index finger abduction movements to 20 degrees while keeping the other fingers still and in a neutral posture. We found that across three participants and two applications per participant, the sensor variation only increased 0.2 degrees during ad/abduction movements compared to a pre-movement rest period, indicating a minimal influence of off-axis movements (Figure S4).

### 4.4. On-Hand Testing of IMUs

We next sought to add additional sensors to our system that could accurately track wrist flexion and extension. We chose to use two IMUs first because they have been used in the prior literature to track bend angles, and second because they can track three angular DOFs, allowing for future work to extract wrist ad/abduction and pronation/supination without adding any additional sensors.

The previous literature has used the same IMUs that are in our system (BNO055) to track movements and has characterized the benchtop performance [14,15] as being <4 degrees of absolute angular error in benchtop tests and 8–15 degrees of angular error in on-hand tests [15]. Here, we sought to validate the on-hand IMU accuracy in our own system with a focus on wrist flexion/extension (Figure 4B,E). The same four participants as in the on-hand strain sensor testing wore two IMUs: one on the dorsal aspect of their palm and one embedded in a smartwatch case (Figure 4E). The relative angles between the two IMUs were calculated using an approach that accounted for slight misalignment/misplacement of the IMUs (Figure 4A). Participants were instructed to perform wrist extension/flexion movements at different speeds (same speeds as in the on-hand testing of strain sensors), while a high-speed camera captured their movements (Figure 4C,D,F,G). During testing of the fastest speed (“as fast as possible”), Participant 4 (P4) had >50% of the camera data labeled as low-quality (see Section 2.13. Thus, P4 is excluded from the “as fast as possible” condition in Figure 4H,I.

The results revealed an MAE, averaged across movement speeds and participants, of 5.07 deg (mean RMSE = 7.27 deg across participants and speeds) for the first application (Figure 4H) and 8.38 deg (mean RMSE = 10.32 deg) for the second application (Figure 4I). Averaged across both applications and all speeds, the MAE was 6.73 deg, with a corresponding RMSE of 8.80 deg. When the absolute errors from both applications were pooled, 79.4% of predictions were ≤10 deg, 92.7% of predictions were ≤15 deg, and 98% of predictions were ≤20 deg.

Consistent with previous work [15], a linear mixed-effects model revealed a significant effect of increasing angular error with increasing movement speed (Figure 4H,I). Specifically, MAE values were averaged across the first and second sensor applications within each participant and speed condition. A linear mixed-effects model with speed as a fixed effect and participant as a random intercept revealed a statistically significant positive association between the speed and mean IMU MAE (slope = 0.585 deg per speed category, z = 2.018, N = 19, *p* = 0.04, 95% CI [0.017, 1.15]). The effect size was modest, with speed accounting for approximately 11.4% of the variance in the MAE (R^2^ marginal = 0.114, R^2^ conditional = 0.508). Considering this, we also report the error statistics when computed using only the two slowest movement speeds. Averaged across all participants and applications, the MAE was 6.35 deg (8.22 RMSE) and 80% of absolute angular errors ≤ 10 deg and 92% were ≤15 deg.

### 4.5. Fully Wireless System

Having established the accuracy of the strain sensors and IMU sensors in estimating joint angles both on the benchtop and on the hand, we next turned to the issue of developing an entire wireless, wearable system. Our system details are outlined in detail (Supplementary Methods: *Detailed wireless system description*). Briefly, the system consisted of the two strain sensors on the index and thumb MCP joints and two IMUs (one on the hand-back unit and one embedded in the watch case). The hand-back unit was a Velcro patch that allowed for cable management from the strain sensors and for holding the IMU in place (Figure 5A). The watch case consisted of the signal acquisition and power board for the IMUs and strain sensors, a microcontroller with a Bluetooth radio, and a rechargeable lithium polymer (LiPo) battery (Figure 5B). It was designed with ports for magnetic connectors from the IMU and strain sensors, as well as a charging port for the battery. The watch case was made from a hard plastic, but snapped onto a soft silicone “wrist-interfacing unit” that was in direct contact with the wrist to ensure user comfort over long durations of time. The whole watch case and wrist unit were mounted to a soft silicone “slap band” watch strap, enabling easy one-handed application.

### 4.6. Testing System Application in a User Study

To ensure our system could be easily applied and removed, we developed and tested two separate strain sensor adhesive strategies (Figure 5C). One strategy used a double-sided adhesive (“toupee tape”, TT) to fix the strain sensors to the hand while another used a liquid silicone adhesive (“Derma-Tac”, DT). Both strategies were tested and compared in a user study (Figure 6).

In this user study, separate participants (N = 4, P5–P9), were brought to the lab and were guided by the experimenter through an initial application and removal procedure. One “procedure” corresponds to application of one full wearable system to one hand using the TT method, and another full wearable system to the other hand using the DT method. Following the initial application and removal (APP0 and REM0), participants repeated the application and removal procedures three times independently (Figure 6A). We found that with each independent application, participants got significantly faster in their application times for both TT and DT (Figure 6B), and in their removal times for TT but not DT (Figure 6D). When directly comparing the two methods, TT was significantly faster than DT to apply (Figure 6C) and to remove (Figure 6E). User survey results also were significantly higher for TT than DT when rating the ease of application, stability of sensor on the hand, and adhesion of sensor to the hand (Figure 6F). Lastly, when the experimenter was monitoring sensor adhesion failures, DT applications resulted in 4/24 total failures (e.g., sensor falls off) and an additional 4/24 partial failures (e.g., sensor lifts at edge, like what occurred with participant P4, Figure S2), whereas TT applications had 0/24 total failures and 1/24 partial failure (Figure 4G). Overall, we found the TT application method to be much better than the DT method in many respects. Furthermore, the method allowed for full system application in ~5 min and full system removal in ~4 min. 

### 4.7. Full System Demonstration

To demonstrate the potential of the wireless system, one participant wore the system on one hand continuously for ~9 h during activities of daily living (Figure S9), after which recording stopped due to battery depletion. A representative 3 h segment is shown in Figure 7A, with example 20 s activity windows in Figure 7B–G and examples of signal acquisition artifacts shown in Figure S10.

Across activities, Figure 7B–G provides a strictly qualitative demonstration that the measured kinematics were consistent with expected movement patterns. During walking, the index and thumb joint angles remained near baseline while the wrist flexion/extension exhibited low-amplitude periodic oscillations consistent with natural arm swing (Figure 7B). During texting, the index finger and wrist had minimal motion as they remained stationary to support the phone, while the thumb exhibited repeated flexion consistent with typing on a phone (Figure 7C). During typing, the wrist motion remained low due to support from the tabletop, and the thumb motion remained low due to the dominant use of the contralateral hand for spacebar pressing, while the index finger showed moderate modulation corresponding to key presses (Figure 7D). During rest, all joints remained near the constant baseline values with minimal variation (Figure 7E). During piano playing, coordinated modulation of the wrist and index finger was observed and thumb motion remained limited, consistent with the participant’s novice playing strategy (Figure 7F). During eating, movements were slower and less periodic, reflecting a mixture of reach, grasp, and transport actions interspersed with periods of stationary joints due to chewing and swallowing (Figure 7G).

Multiple calibrations were performed throughout the sessions (Figure S9B,C). Calibration curves showed modest variation across time. Note that the ground truth angles were not verified by an experimenter and were just estimated by the participant on their own due to the in-the-wild nature of the data collection. It is thus possible that the variation in calibration curves reflect a combination of minor sensor changes and some uncertainty in manual angle positioning.

In this demonstration, which represents a single observation (N = 1), the battery lasted longer than expected: ~9 h, compared to the conservative battery-life calculations of ~8.2 h in Table S3. The current draw from the XIAO ESP32C6 Development board is an approximation and the current estimates for the BNO055 and MAX6070 are set to the max current draws from the datasheet; for other components, typical values from their datasheets were used. The battery life estimate was calculated from the 680 mAh LiPo battery’s estimated usable energy down to the buck converter’s loss-of-regulation limit at the system’s supply voltage, then accounting for the converter’s efficiency at the expected input/output voltage and load current. For the above assumptions, it is possible that in practice, the current draws are lower than the maximums, the battery may deliver near or above its rated capacity for a low discharge rate, and the device continues to operate below the loss-of-regulation limit, resulting in a longer battery life duration than estimated.

### 4.8. System Cost

Lastly, we present the cost to build a single device across different stages of development (summarized in Table S2). The prototype version used in this study has a per-unit cost of $111 (Table S2, column 2). For future iterations, we plan to integrate all electronics into a custom PCB with improved power management and user controls, bringing the estimated cost to $118 per unit (Table S2, column 3). Finally, scaling up manufacturing quantities to leverage economies of scale is estimated to reduce the cost to $55 per unit (Table S2, column 4). Note that the custom PCB design and scaled manufacturing figures are engineering estimates.

## 5. Discussion

In this study, we present a wearable system that leverages in-lab-fabricated thin and flexible strain sensors, off-the-shelf IMUs (BNO055), custom electronics housed in a custom watch, and a rapid application and removal strategy. The entire system was designed to accurately monitor wrist and finger movements, be ergonomic and unobtrusive in everyday life, be easy for people to learn how to apply and remove quickly, and be low cost. While this work presents a wearable system with a validation of accuracy and useability in small unimpaired user studies, future studies in larger populations of movement-impaired participants are required to validate the use of this tool for neurorehabilitation applications.

### 5.1. Under-Sensorization

While we only demonstrated sensorization of the index MCP, thumb MCP, and wrist flexion/extension angle, our current electronic system allows us to use up to four strain sensors, and supports streaming of the full IMU data, allowing future work to extract wrist ad/abduction and wrist pronation/supination as well. Further, because the system is modular, it allows for flexibility in which joints the four strain sensors are placed on. Even with these additions, this system is highly under-sensorized compared to full data gloves and other existing works. However, the downsides of fully sensorized systems are reflected in the cost of the system, the bulkiness of the electronics and the wiring, and the time required to wear or apply them.

We hypothesize that our under-sensorized, yet easily worn out-of-lab hand-tracking system can contribute meaningfully to upper limb neurorehabilitation. First, previous studies have demonstrated that wrist and hand joint angles tend to be highly correlated, and most variance of the hand/wrist during everyday tasks of daily living can be captured by a few dimensions [36,37,38,39]. Building on these observations, under-sensorized kinematic tracking systems have shown promise in algorithmically reconstructing the full hand-pose [40]. We are actively exploring how we may use a combination of data collected from occasional in-lab sessions with highly sensorized tracking systems and frequent out-of-lab data collection with our under-sensorized system. The fully-sensorized datasets would provide insight on which joint angles are most predictive of the hand and wrist’s full posture, and could allow us to fit models that estimate posture from just a few sensorized joint angles. These models could be deployed on the under-sensorized system to have estimates of hand posture outside the lab setting. Secondly, since our system is focused on rehab applications and is flexible and modular, it is possible for us to customize strain sensor placement for individual users, and therefore target joint angles that participants are in most need of monitoring. Therapists could decide this based on what movements are being practiced in physical therapy sessions, and our system could be configured to be sensitive to detection of these movements to assist participants in monitoring their use of these movements outside of the lab. A few sensors, in this case, may be all that is required.

### 5.2. Sensor Accuracy Results

Our liquid-metal strain sensors exhibited a benchtop worst-case (misalignment) MAE of 2.31 deg during benchtop testing (mean RMSE = 2.80 deg), with 91.3% of Model 1 predictions exhibiting absolute angular errors ≤ 5°. Notably, this was driven by misalignments of 10 and 20 degrees, which would be very difficult to achieve on the hand without noticing. During on-hand testing, the MAE was 4.91 deg, with a corresponding mean RMSE of 7.30 deg averaged across applications, with 88% of model 1 predictions exhibiting absolute angular errors ≤ 10°. We note that the on-hand results were specifically for the index finger MCP bending and that the on-hand accuracy of the thumb MCP remains uncharacterized.

We find these results to be largely comparable to existing technologies (Table S1). These on-hand error levels are comparable with those reported by studies with sensors mounted on a wearable glove (Khanna, Oppenheim et al. [11] used electromagnetic sensors and reported a mean root mean square (RMS) error of 6.91 degrees, and Park et al. [9] used liquid metal strain sensors and reported a mean error of 4.61 degrees in calibrated and ~10.5 degrees in non-calibrated applications). Our errors are also comparable to computer vision pose-tracking systems (Mulla et al. [19] reports 5–10° RMSE for index MCP movements). The benchtop testing estimates are comparable to other systems that were benchtop tested (Kim et al. [17] use wearable optical strain sensors and report an MAE of 1.63 degrees, Shenoy et al. [15] use wearable IMUs and report an RMSE of 4–7.5 degrees). Two outstanding exceptions are the Tashakori [10] and Lu [22] studies that report very low RMSEs in on-hand testing (1.21 degrees and 3.78 degrees, respectively). Both leverage sensor technologies (stretchable helical sensor yarns and ultrasound imaging, respectively) that route data to large AI algorithms for inference of joint angles, a pipeline that may not generalize well to movement-impaired populations.

Our wrist angle results from two IMUs exhibited a mean absolute angular error of 6.73 deg, with a corresponding RMSE of 8.80 deg, and errors increased with movement speed, consistent with prior reports using the same IMUs [15] and with the mathematics underlying angular orientation estimation: double integration of accelerometer estimates, in which small errors in accelerometry propagate to large positional errors, become especially erroneous when movements are quickly accelerating and decelerating.

In general, moving from testing sensors in well-controlled benchtop settings to testing on-hand introduces errors associated with (1) differences in sensor bending on the hand than on the benchtop and (2) difficulty in quantifying the ground truth finger bend angle. Differences in sensor bending and data collection on the hand versus on the autobender include a reduced consistency in sensor placement, reduced data for the calibration curve when participants cannot bend their fingers to specified angles (e.g., cannot bend all the way to 90 deg), and variability in the participant interpretation of instructions (e.g., place hand flat on the table). Beyond these differences, there are also inherent challenges in quantifying finger bend angles from camera frames, including marking joint locations reliably and off-axis hand rotations.

Overall, even though our on-hand testing was in a small population, we find that our results are in-line with existing on-hand sensor technologies, with the several user benefits outlined below.

### 5.3. Reuse of Calibration Curve for Strain Sensors

First, we found that when a calibration curve was fit from data from one application, it generalized well to a second application if the data was normalized according to data collected in the second application (Figure 3I). This implies that a detailed calibration curve can be developed when participants first apply the sensor, and that when participants apply the sensor each subsequent time (i.e., each application), a quick ~5 s data collection snapshot can be taken for each strain sensor to identify the maximum and minimum ADC values achieved when the user sweeps through their full range of motion. While initially promising, follow-up study is required to confirm the validity of this strategy in a larger population and in movement-impaired populations.

### 5.4. Monitoring of Data Acquisition

Our priority in the layout of the custom signal acquisition PCB was minimizing the board area and minimizing power consumption to maximize the wearable battery life. To achieve this without compromising data quality, we opted for a software-based calibration routine rather than continuous hardware-level self-monitoring loops. This trade-off significantly reduces the component density and active power draw. Another approach toward ensuring good data quality is on the software side. If the software acquiring data is given access to a joint probability model across monitored joints, it could detect and notify the user about measured points with low probability. However, for continuous-wear scenarios where component degradation and trace aging can impact circuit reliability, incorporating low-overhead embedded optimization and hardware-level validation frameworks (e.g., [41]) represents an important approach toward achieving fully autonomous, robust data acquisition in future iterations.

### 5.5. Cost of System

Second, few studies report on their final cost, but here we intentionally developed a low-cost system (Table S2). We emphasize cost because feasibility for routine rehabilitation use depends on scalability and replaceability, in addition to accuracy. Detailed cost reports are not typically reported in academic studies of wearable kinematic monitoring systems, but costs of upwards of $1000/glove are common amongst highly-accurate commercial data gloves. Examples include the Manus Metagloves Pro, a wireless glove tracking 25 degrees of freedom, which costs >$5k without any license for software. StretchSense offers a fingertipless glove with 32 tracking sensors for $495 as their lowest level product, with offerings of motion capture gloves costing up to $2995 (no software included). Rokoko offers a Smartglove for motion capture for $1995, and Noitom offers a motion capture glove for >$2000. Each glove varies in its form factor and its intended use—many are for motion capture for creating animated movies or interacting with video games.

### 5.6. Application and Removal of System

Beyond accuracy, long-term rehabilitation utility depends on independent donning/doffing and safe storage that supports frequent reuse without damage. A major focus of this work was therefore the development of a user-friendly application and removal strategy. Few wearable sensing studies report use outside of controlled laboratory settings, and even fewer describe how sensors are applied, removed, and managed by users without technical assistance. Enabling users to independently apply, remove, and store these systems is critical for translating wearable technologies from lab-based demonstrations to real-world rehabilitation.

In our study, users were able to apply the full system in approximately 5 min after minimal practice, with removal taking approximately 4 min. These times reflect a practical setup that is compatible with repeated daily use.

### 5.7. Direct Hand-Mounting

We chose to mount the strain sensors directly on the skin rather than using a glove-based architecture. While data gloves can provide dense sensing coverage, they need to have excellent fit, else sensors will not be aligned to joints. They may also restrict tactile sensation or interfere with other daily tasks. For example, users would be unlikely to eat while wearing expensive data gloves, but were able to do so in our study (Figure 7). Direct on-skin mounting enables a lightweight, low-profile form factor that preserves tactile feedback and naturalistic use of the fingers and palm. It also enables modular sensor placement based on the person and their sensing needs, preventing over-sensorization when it may not be required. This approach also reduces the system cost.

Importantly, direct mounting introduces challenges in consistent placement and variable adhesion, which motivated the development of the alignment guides and application workflow described in this work. Future work will seek to evaluate these application and removal strategies in larger populations of individuals with motor impairments and to further refine methods for improving repeatability, comfort, and long-term usability.

### 5.8. Robustness to Real-World Disturbances

Real-world use introduces environmental and motion-related disturbances that were not fully characterized in the present study. Several results provide preliminary evidence for robustness to movement-related artifacts. Strain sensor error did not significantly increase with MCP flexion/extension speed, and index MCP sensor variation increased by only ~0.2° during off-axis abduction/adduction compared with rest. In addition, the system produced activity-consistent signals during a ~9 h proof-of-concept wear session that included walking, texting, typing, resting, piano playing, and eating. However, these experiments do not fully quantify all expected real-world artifacts, including sensor slip, wire motion, direct contact pressure, transient impacts to the watch enclosure, temperature-dependent drift, or electromagnetic interference.

Direct pressure on the sensor is a particularly important potential artifact source for skin-mounted resistive strain sensors, and in preliminary observations produced high-frequency modulations in the unfiltered signal. This suggests that transient pressure may be distinguishable from voluntary kinematics based on its frequency content. Future work will systematically quantify normal pressure, shear loading, sensor slip, and impact artifacts, and will investigate artifact-rejection approaches.

Temperature-dependent drift and electromagnetic noise may also affect long-duration measurement stability. In our system, the EGaIn channel is embedded within Ecoflex, so the temperature of the liquid-metal channel itself may differ from the ambient, skin, or electronics’ temperature. EGaIn-filled microchannels have temperature-dependent resistance, with prior work reporting a temperature coefficient of resistivity on the order of 10^−3^/°C [42]. This means a temperature change of 30 °F (16.7 °C) in the EGaln channels would correspond to a shift in 0.16 Ohms, which is 2% of our strain sensor resistance, measured as 0.7 Ohms. Based on our recorded ADC data, a resistance change of this magnitude could be a non-negligible fraction of the observed signal range, particularly for small-amplitude movements. Thus, temperature changes could contribute to slow baseline drift during extended wear. In the present system, bend angles are estimated from calibration and normalized sensor values, which may reduce but does not eliminate sensitivity to thermal drift.

We did not observe obvious electromagnetic-noise-related failures during the proof-of-concept long-wear recordings, but these recordings were qualitative demonstrations rather than controlled electromagnetic-interference tests. The current strain sensor readout includes analog and digital filtering, including a first-order passive RC low-pass filter with −3 dB cutoff at 1.6 kHz, and a fourth-order SINC filter (filter word FS = 8) on two enabled channels on the AD7124 ADC. With an intrinsic conversion rate of ~2400 SPS per channel with notches at 2400 Hz harmonics and a −3 dB cutoff at 576 Hz, the channel-switching settling time sets the effective sampling rate as 300 Hz. These filtering stages reduce higher-frequency electrical noise before downstream analysis. These filtering stages are appropriate for hand-kinematic measurements, which occur at relatively low frequencies (typically 0–10 Hz) compared with high-frequency electrical artifacts. Future hardware revisions will use a more integrated PCB layout, shorter interconnects, improved grounding, and shielding/guarding strategies to reduce susceptibility to electromagnetic interference.

### 5.9. Future Directions

Future work involves (1) further development of the strain sensor fabrication process to improve the on-hand accuracy, reduce manual labor, and improve sensor yield, (2) testing and iterating upon our application/removal strategies in larger movement-impaired populations, and finally (3) the deployment of our system in neurorehabilitation contexts.

In our system, the dominant barrier to increased sensor reliability, accuracy, and general scalability of our system is the sensor design and fabrication process. Within the sensor design, sensor geometry improvements could improve accuracy. Liquid-metal microfluidic sensors with Peano-type fractal microchannels have been reported to have lower hysteresis and improved stretchability compared with straight channels. This could manifest as an improvement in the consistency of our sensor readings, particularly at the extreme angles, resulting in more consistent calibration curves. There are also ways to develop sensor geometries with orthogonal channel elements that enable multi-directional strain sensing [43]. While our strain sensors did not demonstrate considerable changes in signals with the index finger abduction/adduction movement (Figure S4), it is likely that joints with a greater range of motion of abduction movements, such as the thumb, may be more susceptible to ‘off-axis’ contamination of sensor readings. Orthogonal channels could help separate MCP bending from abduction. Additionally, as discussed above, applied pressure to the channel due to objects pressing on the sensors would also drive signal changes. Recent work has found that off-axis stretching and applied pressure can be dissociated from on-axis stretching by using AC-enhanced liquid-metal sensing [40]. This technology has been shown to distinguish in-plane stretching from out-of-plane compression by leveraging frequency-dependent effects alongside DC sensing. This type of multimodal readout could reduce ambiguity in on-body measurements when deployed in the real world, especially when contact pressure and joint bending may co-occur. A final sensor geometry improvement could be to increase the number of sensed joints per sensing strip. Prior wearable finger-sensing work shows that meander-pattern strain gauges can separately capture MCP and PIP joint angles using multiple sensing zones on a single, thin, skin-conforming substrate [44]. While that work uses thin-film metallization rather than liquid-metal microchannels, the goal is consistent: supporting more detailed reconstruction of finger posture without adding more bulk.

Advancements in manufacturing processes can enable these design upgrades. Silicone microfabrication approaches (e.g., spin-coating, photoresist-patterned molds, and metal deposition) support fine features and repeatable geometries [43,45]. Direct-ink-writing and dispensing platforms can also accelerate iteration on soft substrates and reduce operator-dependent variability during prototyping [46]. Collectively, these steps could reduce the per-sensor labor cost, improve yield, and decrease sensor-to-sensor variability.

Beyond the strain sensor development, a significant contribution of this work is the application and removal strategies tested and the associated tools. While these strategies were designed to be as user-friendly, quick, and accessible as possible (e.g., a slap-band wrist strap, the use of magnetic connectors on the wrist watch, large tabs on HBU adhesive liners, and a simple folding motion of the TPU required to apply sensors), we have not tested the approaches in a movement-impaired population. Other studies have developed customized application strategies for wearables that include the development of customized hand-molds [30] or printing directly on the skin [46], but these are not scalable nor cost-effective. Future work will seek to make our developed strategies as accessible to the movement-impaired population as possible. We anticipate people with spasticity, upper limb weakness, or tremor may have the most difficulty in application and removal, and would seek to test in and make improvements for this population specifically.

Lastly, as mentioned above, we are actively seeking to test whether our under-sensorization approach may be useful in neurorehabilitation contexts, especially for people with upper limb disability undergoing physical rehabilitation. We hypothesize that providing patients with feedback about their hand use during everyday life could help motivate (1) more integration of the hand into daily tasks generally, and (2) adherence to the assigned exercises that are part of physical therapy. Examples of useful metrics to display to patients might be what their ranges of motion for individual joints are [11], how much variance there is in individual joints over the course of the day, indicating how often joints are moved, and how consistent joint movements are during repeated movements [11], as well as movement classifications to indicate what types of movements have been done. In the current system, joint angles are estimated using a deliberately simple calibration and normalization pipeline. However, future versions could incorporate AI-enhanced and embedded signal-processing strategies to infer these metrics from under-sensorized longitudinal recordings, improve robustness, and personalize feedback to individual users. Recent reviews of AI-driven wearable electronics highlight the role of AI in wearable signal interpretation, personalized monitoring, human activity recognition, and real-time health analysis [47,48]. In our system, similar approaches could be used for activity classification, user-specific and adaptive recalibration, artifact detection, sensor-failure detection, and the estimation of richer hand-use metrics from an under-sensorized device. Embedded or edge-AI implementations could also allow monitoring and failure detection to run locally on the wearable or paired mobile device, reducing continuous raw-data streaming and supporting lower-latency, privacy-preserving feedback [49]. Future work will determine how many joints need to be sensorized to derive these metrics reliably, which algorithms are robust enough for real-world deployment, and how to present data to participants in a motivating, informative manner. Regardless of the specific approach, future work will require larger clinical studies with movement-impaired populations to formally evaluate the system’s clinical utility.

Altogether, we have created a low-cost, modular, under-sensorized hand/wrist monitoring system. We have demonstrated the on-hand accuracy of our system, developed and tested application and removal strategies, and demonstrated initial steps towards ‘in-the-wild’ capabilities, all towards making this device user-friendly and feasible to use outside of lab environments.

## Data Availability

The data presented in this study are available from the corresponding author upon reasonable request. Because some participants did not consent to public release of their photo/video data, only data for which participants granted permission on their Media Records Release Form will be shared.

## Supplementary Materials

The following supporting information can be downloaded at https://www.mdpi.com/article/10.3390/s26154795/s1. Reference [50] is cited in the supplementary materials.
