# Supplementary material for: A Low-Cost, Accurate, and Easily-Worn E-Skin and IMU Hand Kinematic Measurement System
Source: Sensors (Basel). 2026 Jul 28;26(15):4795. doi: 10.3390/s26154795 (PMC13469233; doi:10.3390/s26154795)
Supplement: Supplementary file 1 [file sensors-26-04795-s001.zip › sensors-4362558-supplementary.pdf]

Table of Contents

Supplementary Methods *2*

Strain sensor fabrication post-processing 2

Strain sensor data acquisition circuit 2

IMU communication with microcontroller 3

Wrist flexion/extension calculation details and post-processing 4

Detailed wireless system description 6

Application of the watch, HBU, and IMU 8

Strain sensor donning and doffing 8

Strain sensor donning and doffing components 8

Strain sensor attachment methods 10

Supplementary Figures *11*

Figure S1. Strain sensor data acquisition circuit 11

Figure S2. Anatomical landmark placement on radial aspect of hand and forearm for angular motion-tracking with video 12

Figure S3. P4 Lifting strain sensor characterization. 13

Figure S4. Strain sensor during ad/abduction without any MCP bending 14

Figure S5. DT sensor TPU applicator design with labeled features. 15

Figure S6. TT sensor TPU applicator design with labeled features. 16

Figure S7. TT TPU transfer frame 17

Figure S8. TT transfer workflow using TT TPU transfer frame. 18

Figure S9. Long-wear recording and repeated calibration checkpoints. 19

Figure S10. Representative signal loss and system-level interruptions during extended wear. 20

Figure S11. IMU data acquisition pipeline 21

Figure S12: Strain sensor data acquisition pipeline 22

Figure S13: High level overview of full wireless system electronics 23

Supplementary Tables *24*

Table S1: Comparison of strain sensor and IMU error to existing literature 24

Table S2: Cost estimates of current system prototype and future large-scale batches 25

Table S3: Current draw estimate 27

Supplementary Files *28*

1. Participant Questionnaire 28

2. Test administration note taking sheet 39

Supplementary Methods

Strain sensor fabrication post-processing

Two 28 AWG insulated wire leads (Fermerry) were inserted into the ports of the strain sensor and secured with a small amount of Sil-Poxy™ silicone adhesive (Smooth-On), which also sealed and mechanically reinforced the interfaces. These leads were bundled approximately halfway along their length using a short helix of loop-side Velcro wrap (VELCRO® Brand) for wire management, and a two-pin magnetic connector (JHYOSSTHI) was soldered to the free wire ends to interface with the watch module. For selected sensors that used the toupee tape (TT) method to be applied to the hand (described later), fabric-tape anchors (Micropore™ Paper Tape, 3M) were bonded to the sensor back surface with Sil-Poxy™ to provide an enlarged adhesive footprint. Specifically, the proximal (towards back of hand hand) adhesive contact region used a 25 mm × 12.5 mm tape pad, which covered 12.5 mm at the sensor end and extended beyond the sensor width (11 mm) by 7 mm on each side (14 mm total overhang) to increase effective adhesion area where additional surface area was available. The distal (towards finger) adhesive contact region used a 16 mm × 12.5 mm tape pad with reduced lateral overhang due to the smaller available finger surface.

Strain sensor data acquisition circuit

Autobender

The autobender electronics (Figure S1A) were powered by an Arduino Uno R3, which supplied 5 V to a 3.3V, 800mA linear voltage regulator (LD11173.3, TO220) stabilized by two 10µF electrolytic capacitors. The sensor-reading circuit consisted of a voltage divider configuration in which a 100ohm resistor was in series with the liquid metal sensor (resistance ~0.7 ohms). The voltage between the resistor and the sensor was amplified using an LMC648 op-amp configured for 75x gain, and the amplified signal was digitized using an Adafruit MCP3421 sigma-delta 18-bit ADC breakout board (resolution set to 16-bit, Programmable Gain Amplification (PGA) = 2). A 2.048V precision reference (Adafruit LM4040 breakout) was supplied to the MCP3421 AIN− input. The digitized signal was read by the Arduino Uno I2C communication protocol. The ungained sensor voltage was reconstructed using:

$$V_{ungained} = \frac{(ADCValue \times LSB) + V_{ref}}{Gain_{opamp}} \qquad \text{(S1)}$$

where LSB is the ADC's least significant bit $(31.25\mu V)$, $ADCvalue$ is the value read out from the MCP3421 which varies between -32768 and 32767, V_{ref} is the voltage reference going into the AIN- of the MCP3421 (2.048V), and the $Gain_{opamp}$ is the Opamp gain.

To calculate $\frac{\Delta R}{R_0}$:

$$\frac{\Delta R}{R_o} = \frac{\Delta V_{ungained}}{V_{init,ungained}} \qquad \text{(S2)}$$

where $V_{init,ungained}$ is the ungained voltage measured when the sensor is in its initial unstretched state, and $\Delta V_{ungained} = V_{ungained} - V_{init,ungained}$.

Estimates of $\frac{\Delta R}{R_0}$ were used to compare collected strain sensor data to the physics model predictions (equation 1). Otherwise, ADC data was not used to reconstruct $V_{ungained}$ and was instead normalized between 0 and 1 according to the following expression:

$$ADC_{i,norm} = \frac{ADC_i - ADC_{min}}{ADC_{max} - ADC_{min}} \qquad \text{(S3)}$$

where ADC_i is the sensor value retrieved from the MCP3421 at timestep *i*, ADC_{min} is the minimum observed ADC value and ADC_{max} is the maximum observed ADC value. Normalization was always done when the data being normalized included sensor bending from zero to 90 degrees such that ADC_{min}corresponded to the sensor value when bending was zero degrees and ADC_{max} corresponded to the sensor value when bending was at 90 degrees.

On-hand study:
Because a simple voltage divider cannot support multiple sensors without cross-coupling, and because we sought to minimize current draw from each strain sensor to prolong battery life, for the on-hand study we moved away from the voltage divider circuit (Figure S1B). Instead, an Analog Devices 3.3V LT3045 evaluation board supplied four Analog Devices LT3092 programmable current sources set to 7.5 mA. Each LT3092 excited a sensor beginning at approximately 0.7 ohms with a 2 ohm in-series resistor. The high side of each sensor was amplified using Texas Instrument's OPA333 opamps (gain = 69×). Outputs were digitized using the Analog Devices AD7124-8PMDZ Evaluation Board ADC (PGA = 2) with a 2.048V LM4040 reference on AIN+. Data acquisition was performed with a Raspberry Pi 4 running Kuiper Linux and reading data from the AD7124 via SPI.

Wireless system:
An overview of this system is illustrated in Figures S13. A custom 4-layer PCB with dimensions 3 cm x 2.3 cm was designed to acquire data from up to four channels of strain sensor in a compact form factor. The board takes an external 3.3V voltage input from an off-board Seeed XIAO ESP32C6 development board and powers all components on the board. Similar to the configuration in the on-hand study, four LT3092 programmable current sources are available and set to supply 7.5mA. Solder jumpers are placed for the second, third, and fourth channels of the current sources, so that they can be configured off for unused channels to conserve power; two channels are configured active in our device. Each LT3092 excited a sensor beginning at approximately 0.7 ohms with a 2 ohm in-series resistor. The high sides of each two sensors were amplified using Texas Instrument's OPA2333 dual op-amps (gain = 69×). 1.2 kHz passive low pass filters are applied at each input to the ADC. The AD7124-8 with PGA=2 is used to digitize acquired signals using a 2.5V MAX6070 reference on AIN+ channels. Digitized signals from the AD7124 are communicated to the ESP32 via SPI, which is then streamed via Bluetooth Low Energy (BLE) to a web application on a host computer.

IMU communication with microcontroller

In our device, the IMU is configured to stream data to a host microcontroller. Standard I2C communication is selected by pulling down the IMU protocol select (PS) pins on the PCB, and a 2-way solder jumper is used to select one of the two available I2C addresses (0x28: pull down; 0x29: pull up) upon board bring-up. Two BNO055s in our system are configured with different addresses and share the same I2C bus. SDA and SCL pins of the I2C protocol, along with 3.3V (which powers the sensor board) and GND lines, are made available via four through-holes along

a short edge of the board for connecting to a microcontroller with flexible wires. Prototypes were also made with FFC and JST connectors; the through-hole connection variant was ultimately used in the current iteration of the device because it allows the most flexibility in experimenting with wire properties and hand-attachment strategies to minimize obstruction to daily living tasks, and exhibits the flattest profile. SDA and SCL pins are pulled up to 3.3V with 10kΩ resistors on the PCB, and a solder jumper is selectable for adding a parallel 3kΩ pull-up resistor to the SDA line, which was designed to mitigate the sensor's SDA rising speed issues reported by some of the electronics design community, but has instead proven useful for communicating with the BNO055 over wires up to 2 feet long in some experiment settings. The BNO055 PCB successfully communicates with a Raspberry Pi Pico W and Seeed XIAO ESP32C6 development boards with the default pull down configuration on SDA and SCL pins across multiple prototypes.

Upon initialization by the microcontroller, the BNO055 operates in NDOF (Nine Degrees of Freedom) fusion mode and can be polled at up to 100Hz. Quaternions and Euler angles are streamed from each sensor, and heading is sign-corrected at the firmware level to reconcile coordinate frame convention with the physical mounting orientation. Additionally, a quartz crystal is used to provide a more accurate 32.768kHz oscillation compared to the internal oscillator, and an LED can be enabled by a solder jumper for a power-on indication.

Wrist flexion/extension calculation details and post-processing

We estimated wrist flexion/extension from the IMU placed on the dorsal aspect of the palm (hand-back unit, see below) and the second placed on the dorsal aspect of the forearm inside the watch case (Figure 4E). Each IMU provided a time-varying orientation quaternion $q(t) = [w(t), x(t), y(t), z(t)]^T$, representing the rotation of that IMU from a global/world frame to the local sensor frame. We denote the quaternion from the watch case as $q_{fix}(t)$ and from the dorsal aspect of the palm as $q_{mov}(t)$, respectively. Our goal was to find the rotation needed to go from $q_{mov}(t)$ to $q_{fix}(t)$, along a particular axis that we knew was aligned with wrist flexion/extension (Figure 4A).

Each quaternion was first normalized to unit length and converted to a 3x3 rotation matrix, $R_{fix}(t)$ and $R_{mov}(t)$, using the standard quaternion-to-rotation-matrix mapping [50]. These matrices describe the orientation of the fixed and moving IMU frames with respect to the global frame. The relative rotation matrix to go from the fixed IMU (on the forearm) to the moving IMU (on the hand) was then computed as $R_{rel}(t) = R_{fix}(t)^T R_{mov}(t)$. We next defined two-unit vectors in the local sensor frames, $e_{fix,local} = [1, 0, 0]^T$ and $e_{move,local} = [1, 0, 0]^T$. These vectors approximate the anatomical axis along which wrist flexion/extension occurs (step 1 in Figure 4A). Correct placement of the IMU and orientation of the IMU's x-axis along the flexion/extension axis ensures this alignment.

The vector $e_{move,fixed-frame}(t) = R_{rel}(t) e_{move,local}$ expresses $e_{move,local}$ in the fixed IMU coordinate frame (forearm IMU reference frame), allowing the two vectors $e_{move,fixed-frame}(t)$ and $e_{fix,local}$ to be compared directly for joint angle calculation using a dot product (step 2 in Figure 4A).

The angle between $e_{mov,fixed-frame}(t)$ and $e_{fix,local}$, computed via the dot product, reflects a combination of flexion/extension and abduction/adduction motion. We isolated the flexion/extension component by projecting $e_{mov,fixed-frame}(t)$ onto the XZ plane of the fixed IMU, which corresponds to the plane in which bending occurs for flexion/extension (step 3 Figure 4A).

Specifically, the projection of $e_{mov,fixed-frame}$ onto the XZ plane of the fixed IMU (IMU on palm, Figure 4A step 3) was computed as:

$$e_{move,fixed-frame,proj-XZ}(t) = e_{mov,fixed-frame} - \left(\frac{e_{mov,fixed-frame} \cdot n_{XZplane}}{n_{XZplane} \cdot n_{XZplane}}\right) n_{XZplane} \quad \text{(S4)}$$

where $n_{XZplane}$ is the normal vector to the XZ plane of the fixed IMU. The wrist flexion/extension angle was then computed as the angle between $e_{move,fixed-frame,proj-XZ}(t)$and $e_{fix,local}$.

Because the IMUs could not always be mounted in a perfectly flat orientation on the forearm and palm, a small constant offset was often present in the raw IMU-derived wrist bend angle, even when the wrist was known to be in a neutral 0 degree flexion/extension posture. To correct for this, a baseline offset was computed for each trial as the mean bend angle over the first 40 samples, during which the hand and forearm were stationary and in a zero degree flexion/extension posture. This baseline value was then subtracted from the entire IMU bend-angle time series for that trial.

Occasional discontinuities in the IMU-derived wrist flexion–extension angles were observed due to representational discontinuities in the quaternion-based orientation estimates reported by the BNO055 sensor. The sensor outputs orientation as unit quaternions, for which the numerical representations q and $-q$ correspond to the same physical orientation. For example, in trial 2 of participant P2 during fast movement, the palm-mounted IMU occasionally reported a quaternion of $[w, x, y, z] = [0.81,0.09,0.58,0.02]$, followed in the next time step by its negative counterpart $[-0.81, -0.09, -0.58, -0.02]$, despite no observable change in the physical orientation of the hand. At the same time, the wrist-mounted IMU did not exhibit a corresponding sign reversal and instead showed a jump in one or more of the quaternion values (e.g. the x and y value). Together, this produced a large discontinuity from one sample to the next in the computed relative orientation between the two IMUs.

Wrist flexion–extension was estimated by projecting the relative three-dimensional orientation between the forearm and palm IMUs onto a specific bending axis. When the relative orientation changed abruptly due to the combination of a sign reversal in one IMU and a small orientation update in the other, the intermediate body-axis vectors derived from the relative rotation matrix rotated noticeably with respect to the reference bending plane, even though the underlying physical joint configuration remained unchanged. As a result, the dot product between the projected palm-axis vector and the wrist reference axis could change substantially between successive samples. In the example above, the projected axis yielded a dot product of approximately 0.85 with the wrist reference axis prior to the discontinuity (corresponding to a bend angle of 32 degrees), whereas the subsequent sample yielded a dot product of 0.50 (corresponding to a bend angle of 60 deg). This numerical change produced an apparent jump of nearly 30 deg within a single sampling interval. Because the reported wrist bend angle represents a one-dimensional projection of the full three-dimensional relative orientation, such discontinuities did not necessarily result in 180 deg jumps. To prevent these events from influencing assessed accuracy, if the IMU-derived bend angle between consecutive samples exceeded 5 deg, the affected sample was discarded from further analysis. This procedure rejected < 1% of samples from 30, 60, and 120 bpm, and ~15% of samples from 240bpm and “As fast as possible (AFAP)” speed analysis (see below for on-hand testing details).

Detailed wireless system description

Watch case. A watch case was designed to house the strain sensor acquisition circuit, microcontroller, battery, and one of the two IMU sensors in the system that tracks wrist movement. The top part of the watch case is an open-bottom box printed with PLA with dimensions 30 mm x 30 mm x 25 mm, and is sized to allow flexibility of prototyping and rearranging internal electronics. A custom silicone soft wrist-interfacing unit seals the bottom of the box with a snug press fit, and is designed to allow the off-the-shelf “slap band” with silicone surface to bend and conform to the wrist while keeping the inner bottom side of the watch case flat to maintain the seal and stability for internal electronics. It features a channel to snugly house the slap band, and thin channels perpendicular to it along the top surface with thin walls that stretch to provide strain relief to keep the top side relatively flat as the bottom conforms to the varying curvature of the slap band before/while the device is worn. The silicone surfaces of the strain relief unit and the slap band provide sufficient friction with the wearer’s skin to limit sliding of the device. The strain relief unit is manufactured with FormLabs Silicone 40A resin, and the center of its topside (equal to the bottom inner wall of the watch) is reinforced with a stiffener to enhance flatness and aid adhesion of components in the watch. Electronics housed inside the case are connected via soldered wires and secured via insulating adhesives to inner walls of the watch case or to adjacent components. Cutouts were incorporated in the watch case to press-fit a USB Type-C receptacle for charging the battery or flashing the microcontroller. Cutouts were also designed for press fit of magnetic connectors: two female 2-pin magnetic connectors with 2.54mm pitch pogo pin connections were used to connect to the strain sensors mounted on the Hand Back Unit, and a similar 4-pin magnetic connector was used to connect to the external IMU also on the hand back.

To verify thermal safety during extended wear, we monitored the enclosure's wrist-interface surface temperature over an 8-hour continuous bench-top test. Aside from minor ambient-driven fluctuations in the testing environment (e.g., a baseline shift from an initial 74.0 °F down to 68.4 °F before stabilizing), the device maintained a remarkably steady thermal profile, remaining between 68.4 °F and 74.7 °F. This confirms negligible internal heat generation of the watch case component of the wireless system ensuring user comfort and safety.

Hand back unit. The hand-back unit (HBU) consisted of a 5 × 3 cm area of hook-side velcro strips (QTBLY) and double-sided medical adhesive tape, toupee tape (Vapon), for sticking to the hand-back. The HBU served as a mounting point for the IMU PCB that was placed on the back of the hand as well as strain sensor wires that were tacked down on the HBU’s velcro to keep them out of the way of participant’s hand movements. Loop-side velcro (VELCRO® Brand) was secured to the underside of the PCB for affixing to the HBU. Velcro was also applied to strain sensor wires for affixing to the HBU.

Microcontroller. The ESP32C6 was selected for its built-in Bluetooth and Wi-Fi 6 capabilities, low power mode, and ease of development. The Seeed Studio XIAO ESP32C6 development board was used for its compact form factor and built-in battery management. A battery is connected to the development board’s battery input pins and can be charged when the microcontroller is plugged in to a USB-C power supply. The 3V3 and GND pins of the development board are used to power the IMUs and the strain sensor signal acquisition PCB, and SPI and I2C lines are connected from the designated pins on the development board to the signal acquisition board and IMU/4-pin magnetic connector respectively.

IMU Circuit Board. A 9.5 mm x 11 mm PCB (see “IMU hardware”, Figure S11) with the BNO055 is connected to the microcontroller’s I2C pins, and is mounted flat on the stiffener at the bottom of the watch case for proximity to the surface of the forearm. Data is transmitted via I2C to the

microcontroller via short wires soldered at through-holes on the edge of the sensor board. The I2C bus also connects to the 4-pin magnetic connector.

*Strain Sensor Signal Acquisition Circuit PCB***.** A custom 4-layer PCB with dimensions 3 cm x 2.3 cm was designed to acquire data from up to four channels of strain sensor in a compact form factor (Figure S12). Two wires connecting to magnetic connectors are soldered at through-holes at the edge of the board for each active channel to complete the strain sensor circuit with the sensors external to the watch case. Data is transmitted via SPI to the microcontroller via short wires soldered at dedicated through-holes. Circuit details are in “Strain sensor acquisition circuit – wireless system” and Figure S12.

Battery. A 680 mAh rechargeable lithium polymer battery (YDL Battery) was selected to provide about 8 hours of battery life. Its male JST connector is connected to an external female JST socket, the wires of which are then connected to the microcontroller’s battery input pins. The total system current draw was estimated to be approximately 87.8 mA, yielding an ~8.2-hour battery life estimate: ESP32C6 consumes approximately 45 mA; two BNO055 IMUs in NDOF fusion mode draw ~12.3 mA each; AD7124-* ADC draw ~0.93mA; two LT3091 current sources draw 7.5 mA each; MAX7060 2.5V reference draw ~0.26 mA, and the three indicator LEDs draw ~0.65 mA each. The usable battery energy was obtained by integrating cell terminal voltage over extracted charge, using a typical 0.2C discharge characteristic for a 3.7V LiPo cell with ohmic correction to the instantaneous battery current, from 4.18V to the loss-of-regulation point of 3.30V. This yields 664mAh extracted (97.7%) and 2.51 Wh drawn from the battery per full discharge. With the 95% conversion efficiency of the buck converter (from the SGN6029 datasheet), 2.38 Wh is delivered to the electronics, predicting 8.23 h of operation per full charge.

*Firmware***.** The wearable runs custom firmware written in the Arduino IDE. To reduce dynamic power consumption, the CPU was clocked at 80 MHz from the 160 MHz default to reduce core power draw. The BNO055 IMUs were configured to NDOF fusion mode and sampled at 100 Hz via I2C operating at 400 kHz (fast mode) to output absolute orientation as both Euler angles and quaternions. External crystal oscillators were enabled on both IMUs to improve timing accuracy. On each iteration, calibration status was also read. The AD7124-8 24-bit sigma-delta ADC sampled two differential analog channels simultaneously at 300 S/s per channel. The ADC was configured with a SINC4 filter with FS=8, and communication with the ESP32 used SPI at 500 kHz. Data were transmitted wirelessly via Bluetooth Low Energy (BLE 5.0). The BLE link utilizes a BLE 4.2 LE 1M PHY architecture executing 200 notifications per second._Advertising was configured with a 500 ms. While connected and streaming, every 100 ms, the firmware assembled and transmitted a burst of packets with a maximum payload of 180 bytes, starting with one 75B header and IMU packet (containing identifier, packet counter, timestamp, IMU data blocks, and the number of ADC samples to follow), followed by a 31 B ADC sample packet (containing timestamp, sample count, sample values and channel indices). Device-side timestamps were generated using the ESP’s millis() counter, with the timestamp for each header packet captured after both IMU reads completed. ADC per-sample timestamps within each packet were reconstructed by linearly interpolating from the packet’s base timestamp using the nominal ADC sample interval (1.667 ms). The stable indoor connection range was verified up to 25 meters. To ensure data robustness during daily use, sensor disconnections trigger an automatic exponential-backoff reconnection loop (intervals of 2, 4, 8, and 15 s) that preserves local on-disk recording across the dropout.

Web Application. A browser-based data logging application was developed using the Web Bluetooth API, enabling wireless connection to the wearable from any Chromium-based desktop browser without requiring drivers or native software installation. The application parsed incoming

BLE notifications in real time, decoded the binary packet format, and performed clock synchronization. To align device timestamps to the host clock, the web application implemented a minimum-offset clock synchronization estimators so that a sliding window of the most recent 60 host-device time offsets (~6 seconds of data) was maintained, and the minimum offset within the window was used as the estimated device-to-host offset. This minimum-latency approach mitigates variable BLE scheduling jitter (typically ±25 ms) while preserving the fixed one-way propagation delay (~5–15 ms), ensuring that relative timing between packets remained accurate to within the ESP32's counter stability. The estimator also detected device reboots (forward timestamp jumps >5 s) and millis() rollovers (backward jumps >30 s) and reset accordingly. Data were logged to a unified CSV file in which each row contained a host-synchronized ISO 8601 timestamp, the packet counter, IMU orientation (Euler angles and quaternion) and calibration status for both sensors, ADC voltage measurement for each active channel, and an optional experimenter annotation field. During a data collection session, files were auto-saved to the local filesystem every 15 seconds with a 2-minute rolling overlap to prevent data loss at segment boundaries. The application also provided automatic BLE reconnection with exponential backoff to recover from disconnections without interrupting a recording session. Real-time 3D IMU orientation visualization (Babylon.js) and live strain sensor voltage time-series plots (Chart.js) were included for signal quality monitoring during experiments.

Application of the watch, HBU, and IMU

Participants first donned the watch-style electronics housing on the wrist with the magnetic connectors oriented distally (Figure 5C, step 2). Next, the HBU was applied to the back of the hand after cleaning the skin with an alcohol wipe and allowing it to air-dry; the HBU was oriented with the pull tab facing laterally to support later removal without interference during daily tasks. After both strain sensors were applied and their magnetic leads connected (procedures below; Figure 5C, steps 8--9), the IMU was attached to the HBU and all leads were routed and secured on the HBU to minimize slack and reduce cable motion (Figure 5C step 10).

Strain sensor donning and doffing

Strain sensor donning and doffing components

The general approach for applying the strain sensors to the hand was to mark anatomical landmarks on the hand, mount the strain sensors on a piece of transparent TPU (either “DT sensor TPU applicator” or “TT sensor TPU applicator”, apply an adhesive to the strain sensor while on the TPU applicator, and then to transfer the strain sensor to the hand by folding the TPU applicator over the fingertip and aligning the markings on the TPU to the markings on the hand (Figure 5C, steps 4-7). The following skin preparation procedure, skin marking procedure, and TPU pieces were designed to facilitate this process.

Skin preparation and strain sensor placement marking

Before application, both hands were cleaned with alcohol wipes and allowed to fully air-dry. To standardize strain sensor placement over the metacarpophalangeal (MCP) joints, an experimenter marked anatomical landmarks using a skin-safe marker (BIC BodyMark Temporary Tattoo Marker). For each instrumented digit (index finger and thumb), the MCP joint center was identified by observing repeated flexion/extension; once the apex location was visually confirmed, the experimenter marked the location with a dot and then drew an “X” centered on that location (MCP skin X; Figure 5C, step 1). The person applying the system then assumed the posture used during self-application (index: pointed/extended; thumb: resting on a fist), and the experimenter added short perpendicular dashed alignment lines on the dorsal surface to indicate the intended

strain sensor orientation and match the strain sensor TPU applicator centerline dash (Figure 5C, step 1). These markings served as visual guides to support repeatable strain sensor placement across reapplications.

Sizing and alignment with TPU sizing strip
A reusable TPU sizing strip was used to determine where participants should place their fingers on the TPU applicators to ensure that when the TPU applicator was folded over the fingertip, the sensor was placed correctly on the hand (Figure 5C, step 1). With the digit held in the self-application posture (index pointed; thumb resting on a fist), the sizing strip was positioned under the finger and then folded over the fingertip onto the dorsal surface of the finger (without stretching) so that the printed alignment X on the TPU sizing strip coincided with the MCP skin X (Figure 5C, step 1). While maintaining this alignment, the experimenter identified and recorded the distal finger pad/ tip location on the strip.

This procedure defined the "B" distance (Figure 5C, step 1) from the finger pad/tip to the MCP skin X, and ensured it was the same as the "A" distance from where the sensor would eventually be mounted (X on the TPU) to the finger pad/tip. This recorded distal finger pad/tip location was then transferred by the experimenter onto each digit's sensor TPU applicator.

DT and TT strain sensor TPU applicator design and strain sensor mounting
To enable repeatable alignment and convenient handling during self-application and removal, each strain sensor was integrated with a reusable TPU sensor applicator specific to the adhesive condition (DT sensor TPU applicator or TT sensor TPU applicator; Figure 5C; Figure S4; Figure S5). Both applicator types were fabricated from 0.2 mm-thick TPU sheets cut to a 28 x 8 mm outline and included a printed TPU alignment X (intended to align to the MCP skin X after folding), a dashed horizontal centerline through the TPU alignment X to indicate sensor center when the TPU alignment X was occluded, finger-centering tracks to guide finger placement, and a digit-specific finger pad placement indicator defining the distal finger position during alignment.

The TPU sensor applicator also defined the strain sensor's two adhesive contact points (proximal and distal ends of the strain sensor) using printed outlines/windows indicating where skin-adhesive should be applied (DT) or where tape pads should be placed (TT) (location where "adhesive contact regions" are labeled in Figure S5-6C). For handling and storage, the sensor was held to the TPU sensor applicator using low-tack adhesive mounting squares positioned at the adhesive contact regions. These mounting squares served only as a temporary interface to retain/re-capture the sensor on the applicator before application and after removal. Two small hook-and-loop (Velcro) tabs near the proximal end of the applicator provided a temporary fixture for lead wires during placement and removal. When the sensor was worn, the exposed mounting squares on the TPU sensor applicators were covered with wax paper mounting-square liners to prevent unintended sticking and to keep adhesive surfaces clean (Figure S5-6B).

TT TPU transfer frame for tape placement

For the toupee tape (TT) condition, an additional TPU tool (the TT transfer frame) was prepared by the experimenter prior to each participant (Figure S7). This is for transferring the TT onto the sensor while it is still mounted on the TT applicator. The TT transfer frame carried two pre-cut TT liners at a fixed spacing matching the sensor's proximal and distal adhesive contact regions. The exposed and adhesive toupee tape on the transfer frame was covered with removable wax paper liners for handling and was removed only during tape transfer.

Strain sensor attachment methods

Derma-tac (DT) Application Procedure

With the DT sensors attached to the DT applicators, a liquid skin adhesive (Derma-tac™; Smooth-On) was applied to the sensor's two adhesive contact regions (Fig. 5C, DT step 4). DT was applied with a cotton swab in a thin, uniform layer and allowed to cure for at least 2 min prior to skin contact (Fig. 5C, DT step 5). For application, participants rested the target finger on the table with the finger pad positioned at the applicator's finger pad placement indicator and folded the TPU sensor applicator down to contact the skin, adjusting position until the TPU alignment X aligned to the MCP skin X (Figure 5C, DT step 6). Two anchor points defined correct alignment: (1) the finger pad position at the finger pad placement indicator and (2) the alignment of the TPU alignment X to the MCP skin X. Printed centering tracks and the perpendicular dashed skin lines served as optional visual aids and were not required for alignment._Participants then pressed firmly over the DT-coated adhesive contact regions to bond the sensor in a single, non-adjustable press (Figure 5C, DT step 7), connected the magnetic lead, detached the lead from the applicator's temporary Velcro tabs, and peeled the TPU sensor applicator away starting at the proximal end while ensuring the sensor remained adhered to the skin (Figure 5C, step 8). The procedure was repeated for the thumb while maintaining the thumb-on-fist posture used during measurement.

Derma-tac (DT) removal procedure

To remove the sensor, participants aligned the digit to the TPU sensor applicator using the finger pad placement indicator and MCP skin X anchor point. After removing wax paper mounting-square liners from the applicator's mounting squares, participants pressed and massaged the applicator onto the sensor backing to re-capture the sensor onto the applicator. They then disconnected the magnetic lead from the watch case, routed the wire back onto the applicator's temporary Velcro tabs, and removed the sensor from skin by pinching the proximal sensor end and peeling in a controlled motion. Residual DT was removed from the sensor and skin using a DT remover (Derma-tac™ Remover; Smooth-On) applied with a cotton swab, followed by an alcohol wipe to remove remover residue and allow the sensor to dry. Difficult to remove DT residue left exposed adhesive surfaces on sensors and were therefore covered with wax paper to prevent unintended sticking during storage. finally, the HBU and watch were removed. Steps were repeated for the second sensor. Lastly, the HBU and watch were removed.

Toupee Tape (TT) application procedure

TT sensors were adhered to the skin using a double-sided hypo-allergenic, all-purpose, waterproof, medical adhesive tape (TT; Vapon). When TT sensors were attached to the TT applicator, the TT transfer frame was aligned to the TPU sensor applicator using the TPU alignment X and other reference geometry, pressed to laminate the TT adhesive pieces onto the sensor's fabric tape anchors, and then removed. The exposed TT adhesive pieces on the sensor were then covered with removable wax paper liners to prevent premature adhesion. Participants could then perform an additional alignment check and adjustment phase between the sensor's X and the MCP X before bringing the adhesive into contact with the skin (Figure 5C, TT step 5-6). Once aligned, participants removed the wax paper tape liners and pressed firmly over the tape-covered adhesive contact regions to adhere (Figure 5C, TT step 7). Participants then connected the magnetic lead, detached the lead from the applicator's temporary Velcro tabs, and peeled off the TPU sensor applicator by pinching the proximal end (Figure 5C, TT step 8). The procedure was repeated for the thumb while maintaining the thumb-on-fist posture used during measurement.

Toupee Tape (TT) removal procedure
Participants aligned the digit to the TPU sensor applicator using the finger pad placement indicator and MCP skin X anchor point without lifting the finger. They removed wax paper mounting-square liners from the applicator mounting squares and pressed/massaged the applicator onto the sensor to re-capture the sensor onto the applicator. Participants then disconnected the magnetic lead, routed wiring back onto the applicator Velcro tabs and peeled the sensor from skin using a proximal pinch-and-peel motion. Steps were repeated for the second sensor. Residual tape on the skin was removed by peeling. Sensors were stored mounted on their TPU applicators, and the HBU and watch were removed.

Supplementary Figures

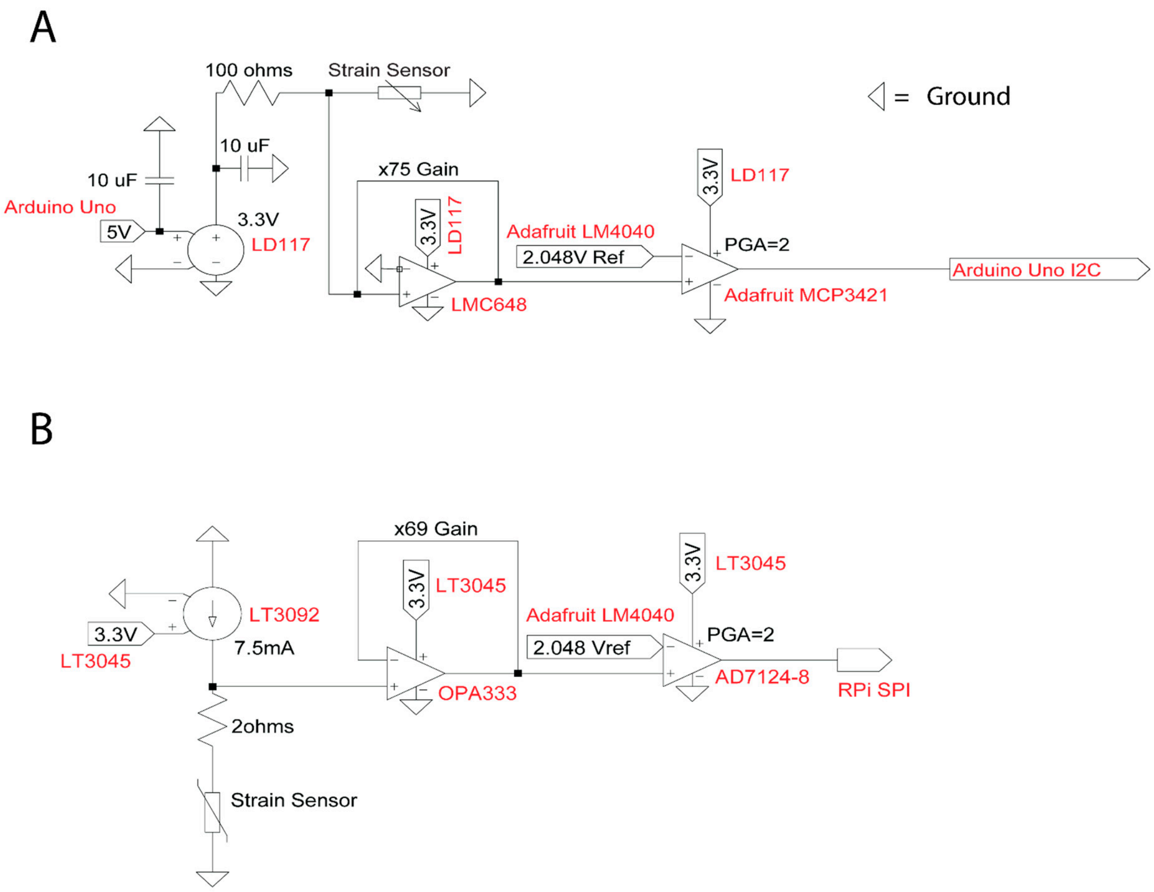

Figure S1. Strain sensor data acquisition circuit

(A) Data acquisition circuit for the autobender **(B)** and on-hand testing

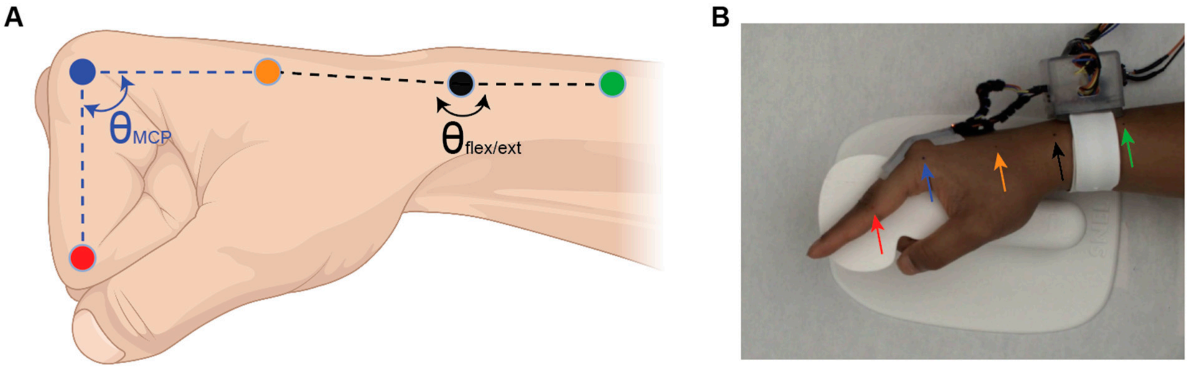

Figure S2. Anatomical landmark placement on radial aspect of hand and forearm for angular motion-tracking with video

(A) Diagram of landmarks and which landmarks are used to calculate index MCP bending and wrist flexion/extension. Partially created in BioRender. Oppenheim, T. (2026). BioRender.com/86pe6ky.

(B) Landmarks on the real hand of one participant

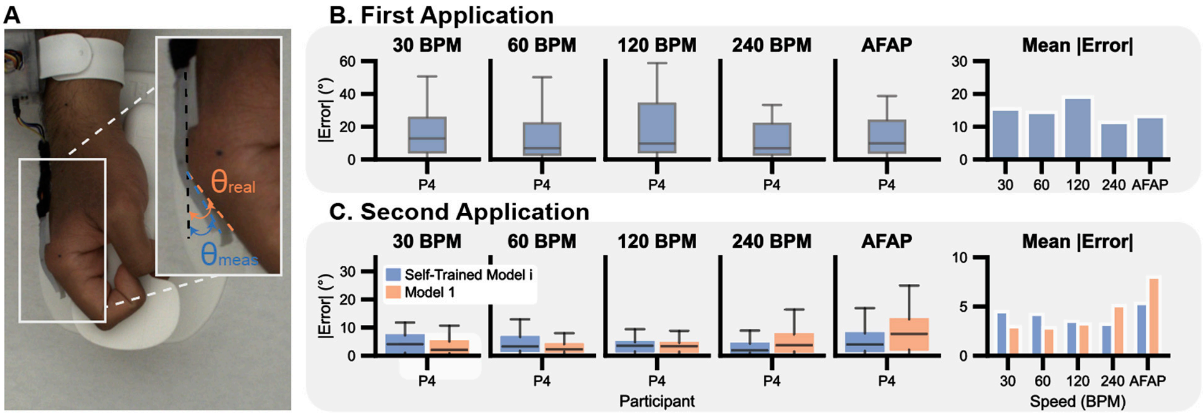

Figure S3. P4 Lifting strain sensor characterization.

(A) Summary absolute angular error box plots from the first sensor application for participant P4 at each MCP bend rate. The bar plot on the far right shows the mean angular error across for P4 at each MCP bend rate.
(B) Second-application summary angular error analysis. Blue boxes and bars represent errors obtained by using the second-application data to develop a calibration curve ("Self-Trained Model i"), while orange boxes and bars represent errors obtained by applying the first-application calibration to the second-application time-series data ("Model 1"). Since the participant's sensor lifting issue was not present during collection of the calibration data, the "Model 1" errors were comparably small to the self-trained model errors. These second application results mirror those of other participants in Figure 3I
(C) Image from overhead camera of participant P4 and their sensor lifting. Θ_real in orange denotes the participant's true index finger MCP bend angle and Θ_measured in blue denotes the angle measured by the sensor due to the sensor lift issue.

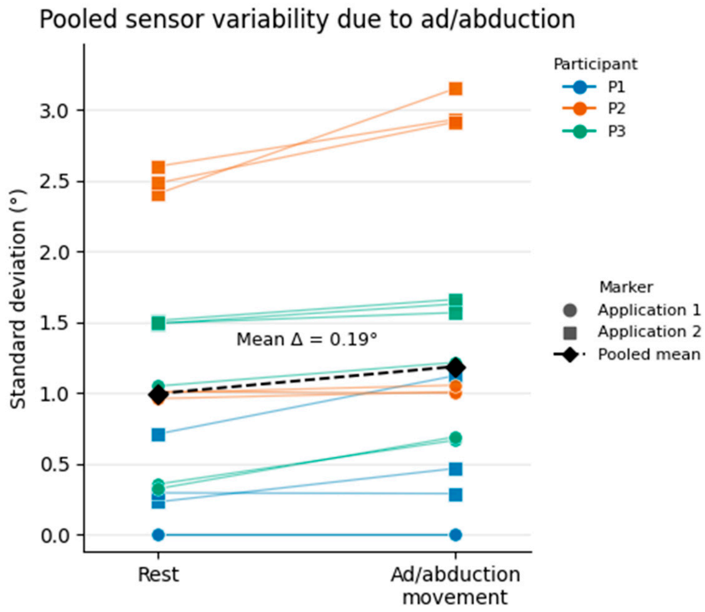

Figure S4. Strain sensor during ad/abduction without any MCP bending

To test how sensitive strains sensors were to movements about the MCP joint that were not MCP bending, we monitored strain sensor signals while participants (N=3, N sensor applications = 2, N trials per application = 3) completed isolated 20 degree abduction movements of the index finger that were verified with captured camera videos. Sensor values (in degrees) were acquired during a 3 second rest period and during the movement period of the abduction/adduction movements. To compare how much sensor values changed during movement compared to rest, we computed the standard deviation of the sensor values during rest versus during movement.

In the figure, each paired line represents one trial. Colors indicate participant, marker shapes indicate sensor application number, and the dashed black line indicates the mean across all participants, applications, and trials. Mean standard deviation increased from 0.996° at rest to 1.188° during movement, corresponding to a small increase of 0.19°.

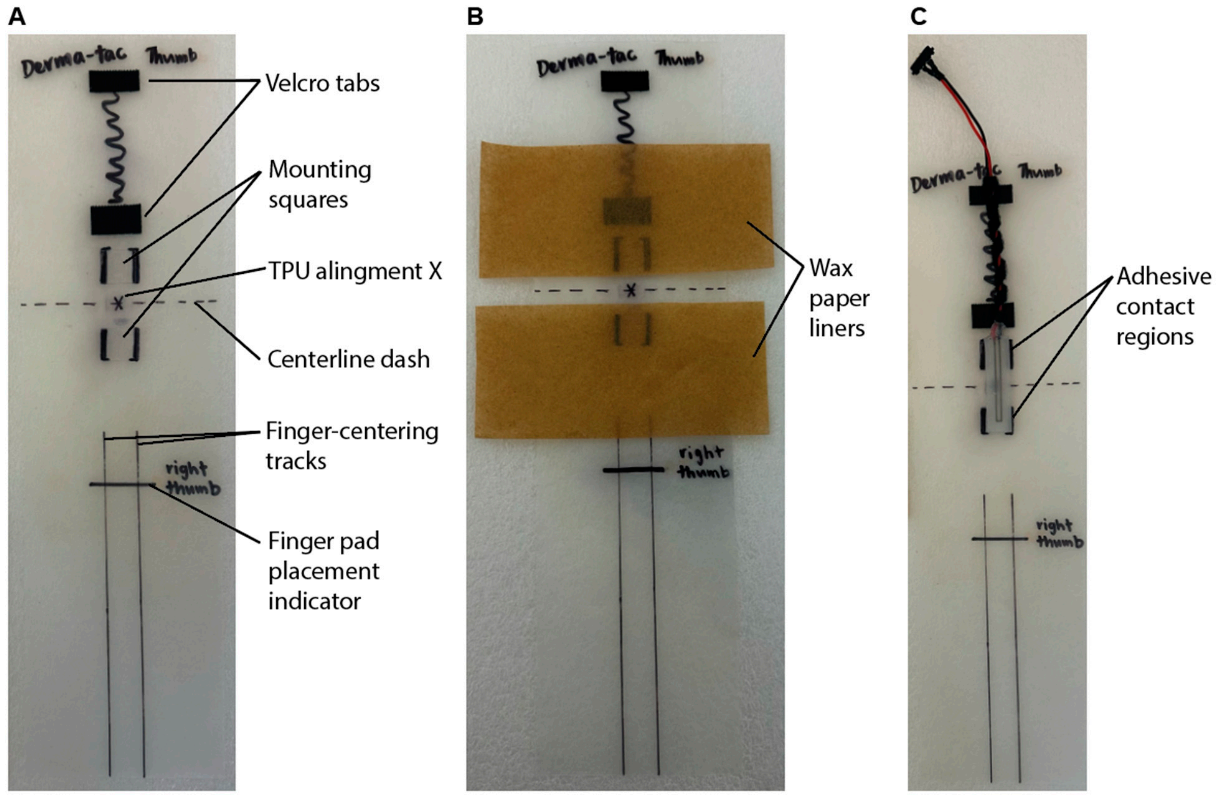

Figure S5. DT sensor TPU applicator design with labeled features.

(A) DT sensor TPU applicator showing Velcro tabs, mounting squares, TPU alignment X, centerline dash, finger-centering tracks, and finger pad placement indicator.
(B) DT sensor TPU applicator with wax paper liners protecting mounting-squares while DT sensor in use.
(C) DT strain sensor mounted on the TPU applicator via mounting squares at adhesive contact regions.

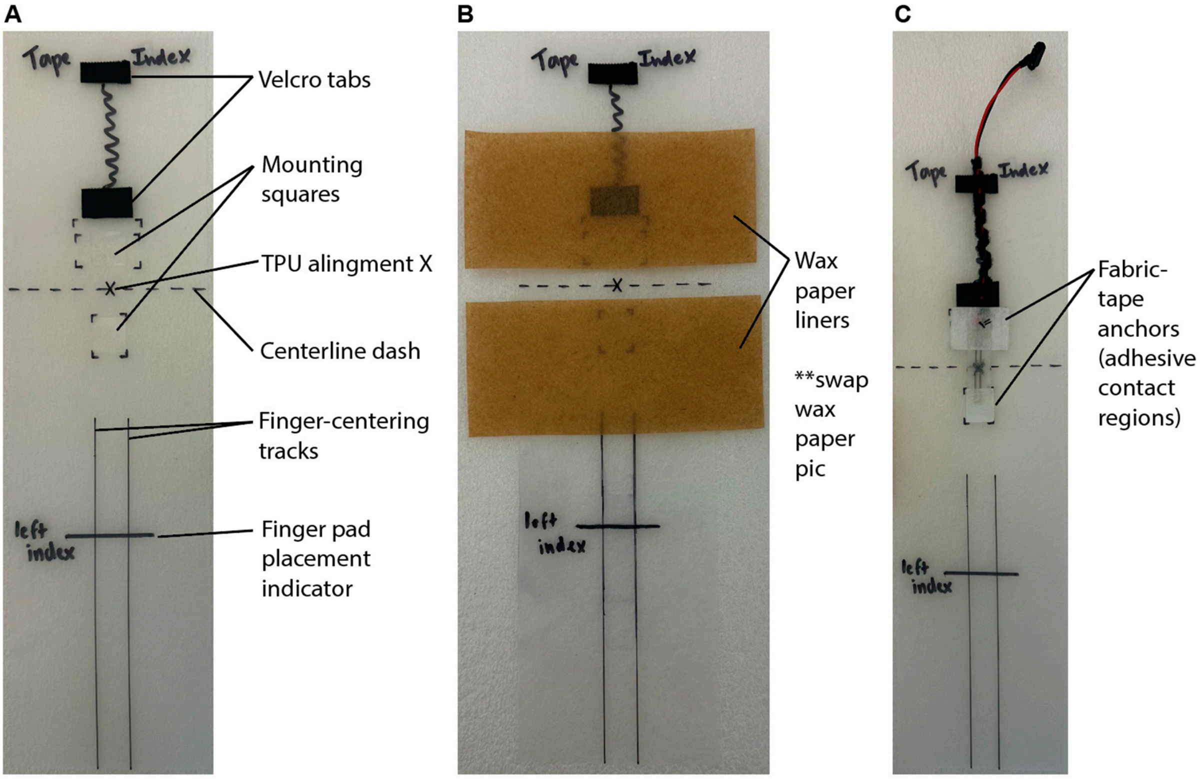

Figure S6. TT sensor TPU applicator design with labeled features.

(A) TT sensor TPU applicator showing Velcro tabs, mounting squares, TPU alignment X, centerline dash, finger-centering tracks, and finger pad placement indicator.
(B) TT sensor TPU applicator with wax paper liners protecting mounting-squares while TT sensor in use.
(C) TT strain sensor mounted on the TPU applicator via mounting squares at adhesive contact regions.

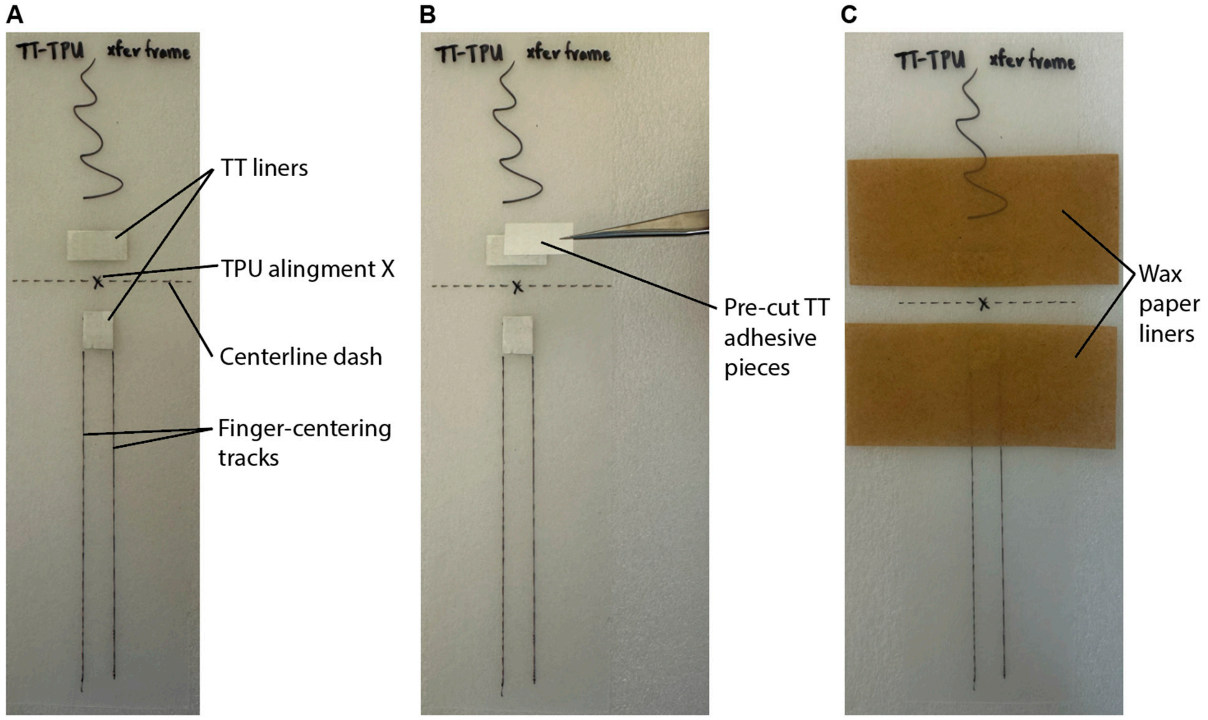

Figure S7. TT TPU transfer frame.

(A) TT transfer frame prepared by the experimenter with two TT liner pieces at fixed spacing matched to the sensor's proximal and distal adhesive contact regions and additional markings (TPU alignment X, centerline dash, finger-centering tracks) to provide optical alignment guides.
(B) Experimenter manually aligning and placing pre-cut TT adhesive pieces onto TT liners.
(C) Exposed and adhesive TT on transfer frame is covered with wax paper liners.

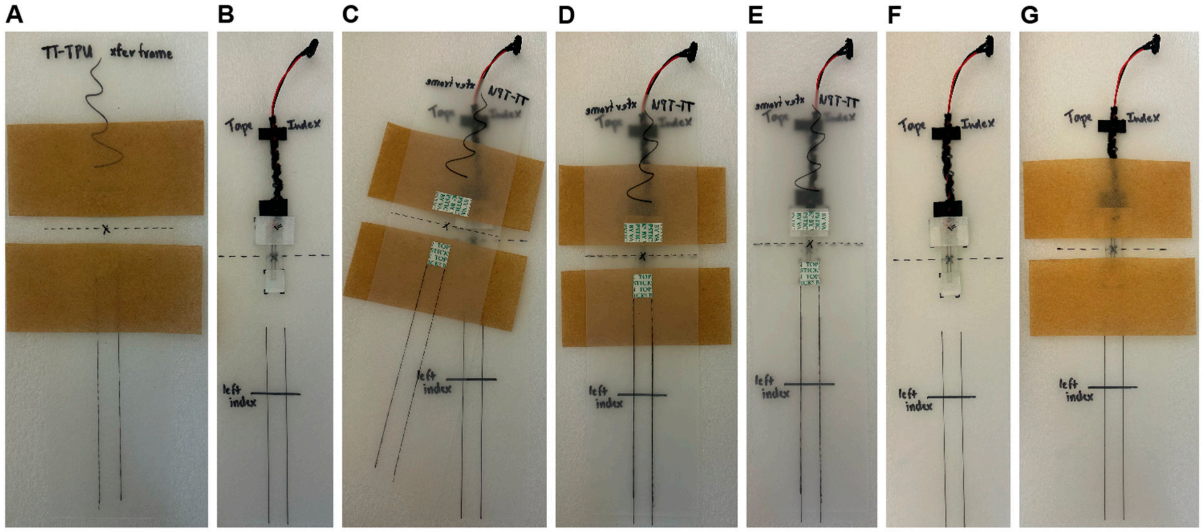

Figure S8. TT transfer workflow using TT TPU transfer frame.

(A) Experimenter-prepared TT TPU transfer frame, as participants received it.
(B) TT sensor TPU applicator, as participants received it.
(C-D) Alignment of TT transfer frame to TT sensor.
(E) Removal of wax paper to expose TT adhesive pieces to sensor's fabric-tape anchors.
(F) TT transfer frame removed, leaving behind TT adhesive pieces on the sensor's fabric-tape anchors, creating the adhesive contact regions.
(G) Replaced wax paper liners on exposed TT adhesive pieces to prevent premature adhesion and enable an additional adjustment phase.

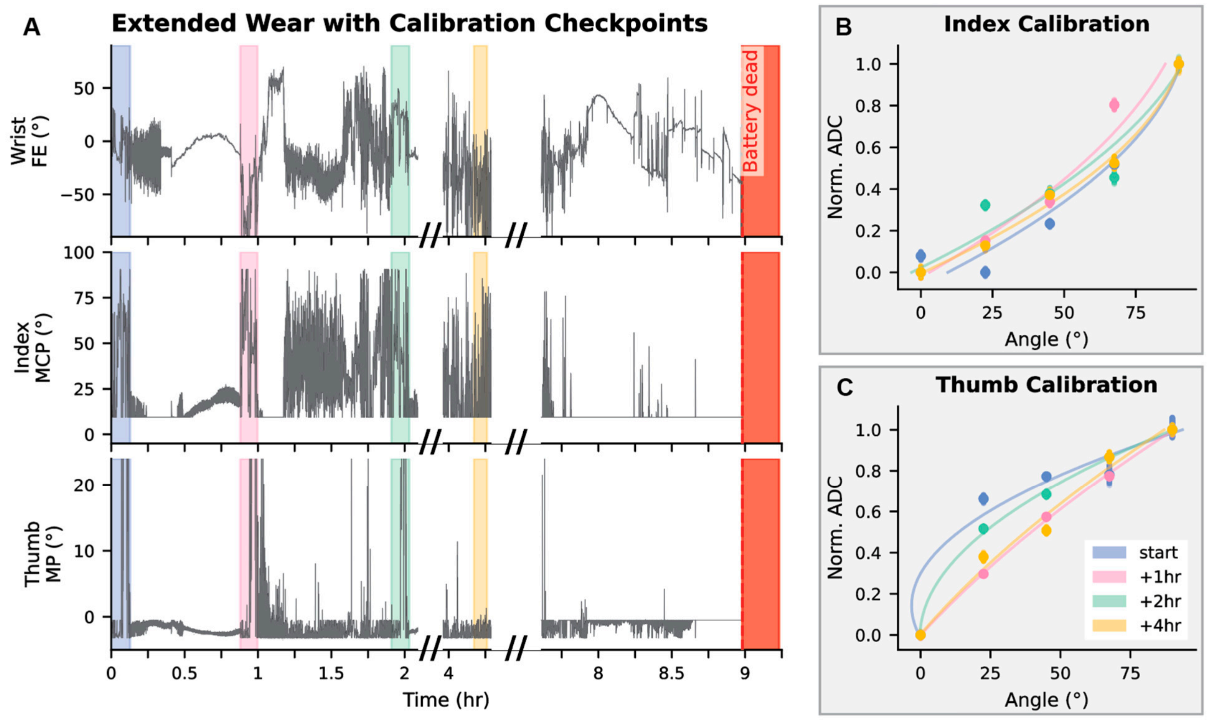

Figure S9. Long-wear recording and repeated calibration checkpoints.

(A) Full 9-hour wear session showing wrist flexion/extension (top), index MCP bend angle (middle), and thumb MP bend angle (bottom). Shaded regions indicate calibration checkpoints collected throughout the session. Recording terminated after approximately 9 hours due to battery depletion.

(B) Normalized calibration curves for the index MCP sensor across calibration checkpoints (start, +1 hr, +2 hr, +4 hr), with points showing normalized sensor values at each target angle and lines indicating quadratic fits.

(C) Normalized calibration curves for the thumb MP sensor across calibration checkpoints.

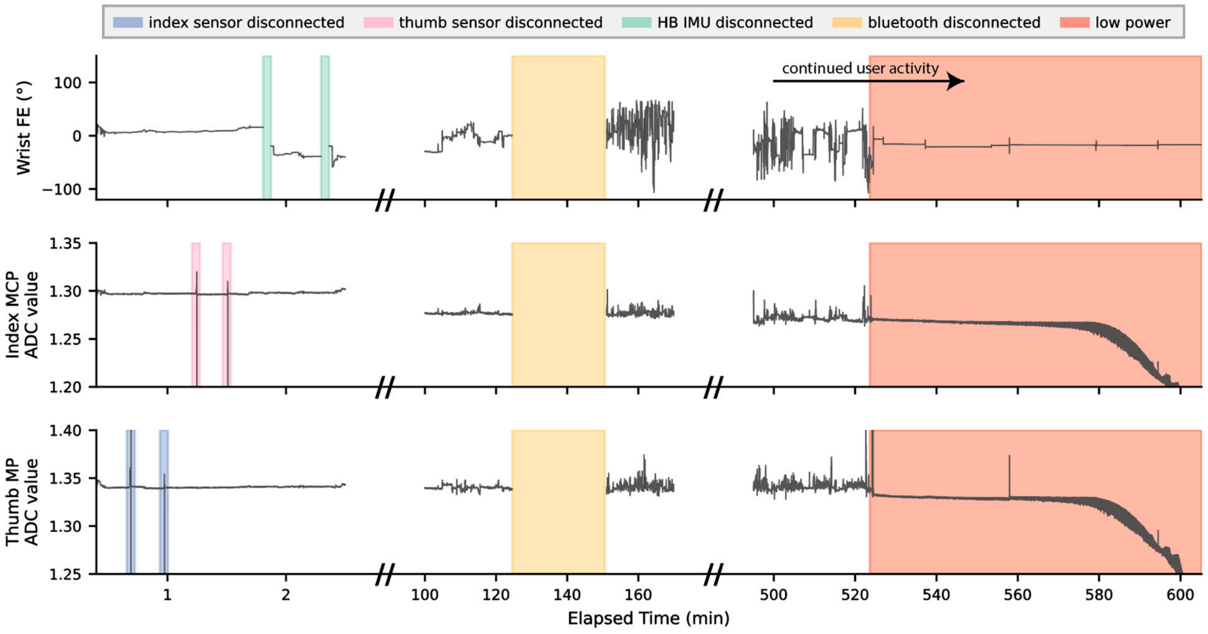

Figure S10. Representative signal loss and system-level interruptions during extended wear.

Wrist flexion/extension (top), raw index MCP ADC value (middle), and raw thumb MP ADC value (bottom) are shown for representative failure modes. The x-axis is not continuous; segments are compressed to highlight distinct events across different time scales. Short-duration, channel-specific disconnections of the index sensor, thumb sensor, and hand-back (HB) IMU are illustrated in blue, pink, and green respectively.

During strain sensor disconnection, raw ADC values rail to approximately −1.75 V. Our post-processing pipeline identifies these events and replaces them with NaNs. HBU IMU disconnection results in interruption of the wrist flexion/extension signal. At the raw signal level, when the IMU disconnects, all four HBU IMU quaternion components collapse to zero for the duration of the disconnection. In our post-processing pipeline, these intervals are detected and replaced with NaNs, appearing here as IMU signal dropout.

A prolonged Bluetooth communication dropout (yellow) produces true signal loss across all channels, resulting in NaN values in the plotted signals. Finally, during the low-power regime (red), signal degradation is observed across all channels despite continued user activity. In the figure above, user activity prior to the red region is the same as user activity after the onset of the red region. Thus, the loss of signal fidelity when the red region begins is due to battery depletion rather than a change in user behavior.

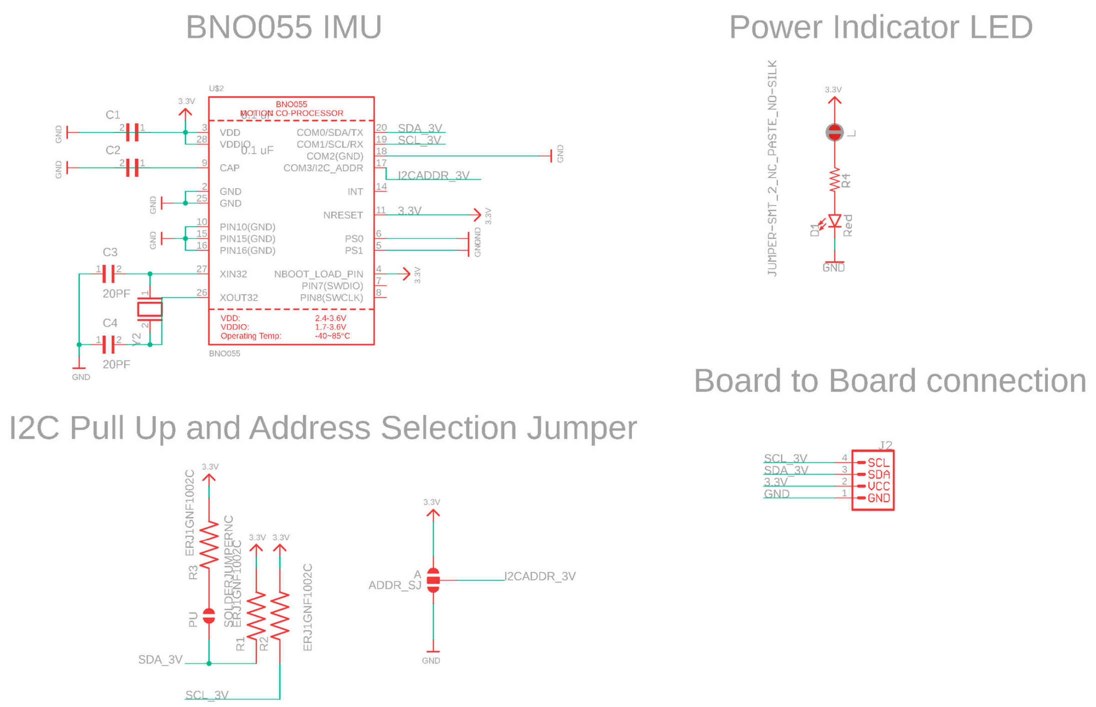

Figure S11. IMU data acquisition pipeline

Parts of the IMU data acquisition pipeline: 1) BNO055 IMU: schematic of the IMU itself with decoupling capacitors (C1-4), 2) I2C pull-up resistors and address selection jumper: pull-up resistors from SDA and SCL to 3.3V, address selection connects the BNO055 address pin to either 3.3V or GND so that two BNO055 sensors can be on the same I2C bus, 3) Power indicator LED provides visual confirmation that the board has power, 4) connection from IMU board to microcontroller.

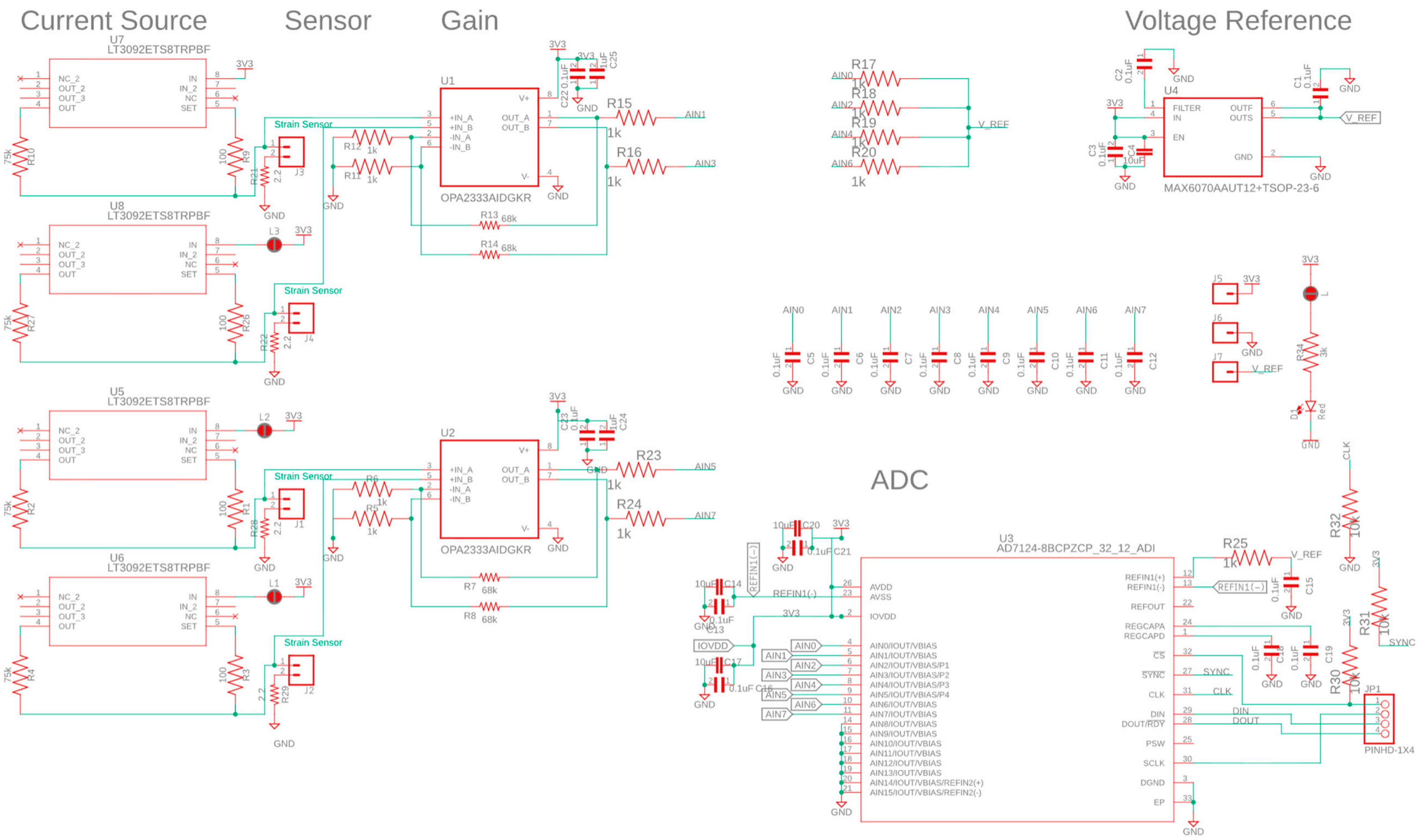

Figure S12: Strain sensor data acquisition pipeline

Parts of the strain sensor data acquisition pipeline used in the full wireless system: 1) current source: four programmable current sources that provide a stable current source to the strain sensors, 2) sensor: strain sensors, 3) gain: voltage signals from the high side of the strain sensor are amplified by an OPA2333 instrumentation amplifier, 4) voltage reference provides stable, high-precision, low-noise voltage reference used in the ADC to reference gained strain sensor voltages against, 5) ADC digitizes data and communicates it to the microcontroller via SPI.

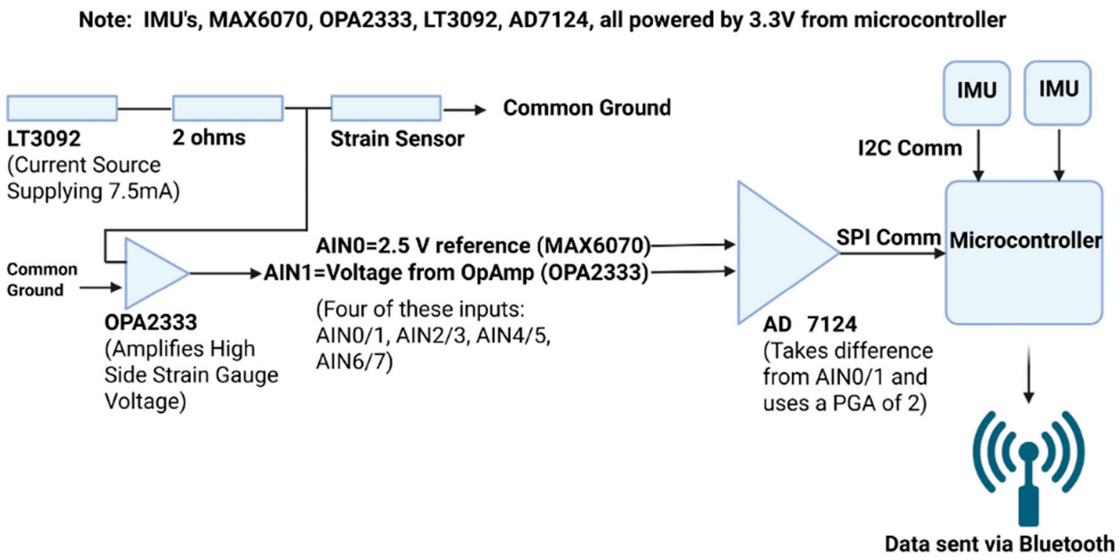

Figure S13: High level overview of full wireless system electronics

Created in BioRender. Oppenheim, T. (2026). BioRender.com/86pe6ky.

Supplementary Tables

Table S1: Comparison of strain sensor and IMU error to existing literature

	Benchtop		On-Hand		
**Architecture**	**MAE (deg)**	**RMSE (deg)**	**MAE (deg)**	**RMSE (deg)**	**Number of participant (N)**
Our Undersensorized LM Strain Sensors	2.31	2.8	4.91	7.3	4
Our Wrist/Palm IMU's: all speeds	-	-	6.73	8.80	4
Our Wrist/Palm IMU's: slow speeds			6.35	8.22	4
Liquid metal strain sensors embedded in stretchable glove (Park et. al. [9], Table 2)			4.51-4.61 (self-calibration) 8.88-11.93 (non-calibration)		13
stretchable helical sensor yarns, IMUs, embedded in a glove (Tashakori et. al. [10] Fig. 3)				1.21	5
Electromagnetic Sensors embedded in a fingerless glove (Khanna, Oppenheim et. al. [11]	-	-	-	6.91	4
IMUs mounted on the hand (Shenoy et. al. [15])	-	~4-7.5	-	8-15	5
Optical strain sensors mounted on the hand (Kim et. al. [17])	1.63	-		-	
Camera Pose-Estimation (Mulla et. al [19])	-	-	-	5 to 10	5
Wrist-mounted ultrasound imaging (Lu et. al. [22])				3.78	8

Table S2: Cost estimates of current system prototype and future large-scale batches

Device Component	Device Subcomponent	Current Version (Qty = 5)	Units per Device	Cost for Specified Quantity	Integrated PCB (Qty = 5)	Units per Device	Estimated Cost for Specified Quantity	Integrated PCB Scaled (Qty = 1000)	Units per Device	Estimated Cost for Specified Quantity
**Note**		This is a prototype version and is hand-assembled in small quantities.			This variant integrates all electronics as a custom PCB and integrates improved power management (for longer battery life) and user controls. Prices for components are recorded on April 21, 2026.			This variant is the same as the "Integrated PCB" version to the left, scaled up in manufacture quantities to estimate the cost advantage of economy of scale. Prices for components are recorded on April 21, 2026.		
Batch Quantity for this variant		**5**			**5**			**1000**		
**PCB Total**				243.79			392.2015			34812.3
PCB Subparts Cost List (First two lines sum to "PCB Total" above; other lines are for clarity)	PCB Bare-board Manufacturing	JLCPCB Bare Board Manufacturing		51.91	Bare Board Manufacturing in China		50	Bare Board Manufacturing in China		300
	Assembly and Parts Total (includes components below in gray)	JLCPCB PCBA		191.88	PCB Assembly in China		342.2015	PCB Assembly in China		34512.3
	Microcontroller	Included below: ESP32C6			ESP32-C6-MINI-1-N4	1	20.1935	ESP32-C6-MINI-1-N4	1	2922.8
	ADC	AD7124	1	52.2235	AD7124-4BCPZ	1	46.2965	AD7124-4BCPZ	1	6494.8
	Current Source	LT3092	4	120.562	LT3092EDD#PBF	4	120.562	LT3092EDD#PBF	4	3904.6
	Amplification	OPQ2333PIDSGR	2	15.664	OPQ2333PIDSGR	2	15.664	OPQ2333PIDSGR	2	1007.6
	Voltage Reference	MAX6070	1	16.6265	N/A (AD7124 Internal Ref)		0	N/A (AD7124 Internal Ref)		0
	On-board IMU (if applicable)	Included with IMU PCB below			BNO055	1	64.15	BNO055	1	8702.9
	Power Management	Included with External Microcontroller Below			MAX77659AENV+	1	38.3355	MAX77659AENV+	1	7279.6
	Indicators and User Interface	Not included			LEDs and Buttons		3	LEDs and Buttons		1000
	Passives	Passives		1	Passives		4	Passives		2500

**Strain Sensor**		Estimate: $3/sensor	2	30	Estimate: $3/sensor	2	30	Estimate: $1/sensor	2	2000
**IMU**		Internal and External BNO055 IMU PCBs	2	139.8583333	Internal, On-board BNO055 (included above with PCB)			Internal, On-board BNO055 (included above with PCB)		
					External IMU PCB	1	69.92916667	External IMU PCB	1	11000
**External Microcontroller (if applicable)**		3 for $13.04 directly from Seeed; $5.38 on Digikey	1	21.6464	Included above in PCB			Included above in PCB		
**Wiring**		Silicone Wires (between boards and connectors)		1	Silicone Wires (to connectors and external sensors)		0.4	Silicone Wires (to connectors and external sensors)		800
**Battery**		YDL 102530 680mAh Battery; $3.17	1	15.85	YDL 102530 680mAh Battery; $3.17	1	15.85	YDL 102530 680mAh Battery; $2.24	1	2240
**Watch Case and Hand Back Unit**	Watch Case	PLA Filament, 13 g, $0.02/g	1	1.3	PLA Filament, 13 g, $0.02/g	1	1.3	PLA Filament, 13 g, $0.02/g	1	260
	Wrist-interfacing unit	Silicone 40A $0.39/ml * 9.2 mL	1	17.94	TPU Filament, 10g, $0.035/g	1	1.75	TPU Filament, 10g, $0.035/g	1	350
	Magnetic Connector 2 Channels	2-channel; Amazon $9.99 for 2 pairs	2	49.95	2-channel; Amazon $9.99 for 2 pairs	2	49.95	2-channel; Alibaba $0.99/pair	2	1980
	Magnetic Connector 2 Channels	4-channel; Amazon $9.99 for 2 pairs	1	24.98	4-channel; Amazon $9.99 for 2 pairs	1	24.98	4-channel; Amazon $0.99/pair	1	990
	Silicone Slap Wristband	Amazon 36 Pcs for $5.99	1	0.83	Amazon 36 Pcs for $5.99	1	0.83	Amazon 36 Pcs for $5.99	1	166
	Hook and Loop (low profile)	Velcro Sleek and Thin $7.93		7.93	Velcro Sleek and Thin $7.93		7.93	Velcro Sleek and Thin $7.93		800
	Toupee Tape	Topstick (Pack of 50) $6.70	2	1.34	Topstick (Pack of 50) $6.70	2	1.34	Topstick (Pack of 50) $6.70	2	160
Total Cost for Qty Specified				556.4147333			596.4606667			55558.3
**Total Materials Cost per Unit**				**111.28**	_	_	**119.29**	_	_	**55.56**

Table S3: Current draw estimate

Component	Quantity	Calculation	Current Draw (mA)	Component Subtotal (mA)
XIAO ESP32C6 Development Board	1	CPU Baseline (80MHz): 15mA | BLE radio avg: 20mA | BLE/I2C overhead: 5mA | LDO & Other: 5mA **= 45mA**	45	45
BNO055 (NDOF fusion mode)	2	Listed in datasheet: NDOF mode max **12.3mA**	12.3	24.6
AD7124-8	1	Listed in datasheet; Full Power Mode typical **930uA**	0.93	0.93
LT3092	2	Set to **7.5mA**	7.5	15
MAX6070	1	Listed in Datasheet: 150uA typical supply current, max Iq **260uA**. Supplies into high Z ADC inputs	0.26	0.26
OPA2333	2	Datasheet: 34uA per two amplifiers typical; 70pA typical input bias	0.034	0.068
Indicator LED	3	(3.3V - 2.0V) / 2kΩ = **0.65mA each**	0.65	1.95
			**Total**	**87.808 mA**
Battery life estimate<br>2.51 Wh usable battery energy (664 mAh × 3.79 V mean, integrated to the ≈3.30 V buck loss-of-regulation limit) × 95% converter efficiency ÷ (87.808 mA × 3.30 V)<br>(using 680mAh 3.7V LiPo cell)				**~8.2 hours**

Supplementary Files

1. Participant Questionnaire

Wearable for Continuous Monitoring of Hand Kinematics: Aim 1 Post-Study Questionnaire

Thank you for participating in our study! Please answer the following questions based on your experience wearing and using the hand tracking device. Your responses are essential in helping us improve comfort, usability, and sensor performance.

Section 1: Handedness

Which of your hands is dominant? (circle one): Right / Left / Neither (ambidextrous)

Section 2: Rating Questions (Scale 1–10)

Please rate the following on a scale of 1 (very poor/disagree) to 10 (excellent/agree strongly).

Long-term Comfort While Wearing: Referring to the first time you wore the device: How comfortable was the device to wear (10 is very comfortable, 1 is very uncomfortable)?

Adhesive: Toupee Tape

Hand worn (circle one): Left / Right

Score ___/ 10

Adhesive: Derma-tac

Hand worn (circle one): Left / Right

Score ___/ 10

Additional Comments: ____

____

____

____

____

Ease of Initial Application **Without** Constraints: How easy was it to apply the entire wearable system for the first time? (10 is very easy, 1 is very difficult)

Adhesive: Toupee Tape

Hand worn (circle one): Left / Right

Score ___/ 10

Adhesive: Derma-tac

Hand worn (circle one): Left / Right

Score ___/ 10

Additional Comments: ____

____

____

____

____

Ease of Reapplication **with** Constraints: How easy was it to remove and then reapply the sensors by yourself while your hand was constrained with splints? (10 is very easy, 1 is very difficult)

Adhesive: Toupee Tape

Hand worn (circle one): Left / Right

Score ___/ 10

Adhesive: Derma-tac

Hand worn (circle one): Left / Right

Score ___/ 10

Additional Comments: ____
____
____
____
____

Ease of Final Application **Without** Constraints: How easy was it to apply the entire wearable system for the second time? (10 is very easy, 1 is very difficult)

Adhesive: Toupee Tape

Hand worn (circle one): Left / Right

Score ___/ 10

Adhesive: Derma-tac

Hand worn (circle one): Left / Right

Score ___/ 10

Additional Comments: ____
____
____
____
____

Freedom of Movement:

To what extent did the devices allow for natural hand and wrist movement? (10 is very natural movements, 1 is very unnatural movements)

First application (applied without constraints)

Adhesive: Toupee Tape

Hand worn (circle one): Left / Right

Score ___/ 10

Adhesive: Derma-tac

Hand worn (circle one): Left / Right

Score ___/ 10

Second application (applied with constraints)

Adhesive: Toupee Tape

Hand worn (circle one): Left / Right

Score ___/ 10

Adhesive: Derma-tac

Hand worn (circle one): Left / Right

Score ___/ 10

Final application (applied without constraints)

Adhesive: Toupee Tape

Hand worn (circle one): Left / Right

Score ___/ 10

Adhesive: Derma-tac

Hand worn (circle one): Left / Right

Score ___/ 10

Stability During Motion:

How stable did the whole system feel during movement and physical tasks? (10 is very stable, 1 is very unstable)

First application (applied without constraints)

Adhesive: Toupee Tape

Hand worn (circle one): Left / Right

Score ___/ 10

Adhesive: Derma-tac

Hand worn (circle one): Left / Right

Score ___/ 10

Second application (applied with constraints)

Adhesive: Toupee Tape

Hand worn (circle one): Left / Right

Score ___/ 10

Adhesive: Derma-tac

Hand worn (circle one): Left / Right

Score ___/ 10

Final application (applied without constraints)

Adhesive: Toupee Tape

Hand worn (circle one): Left / Right

Score ___/ 10

Adhesive: Derma-tac

Hand worn (circle one): Left / Right

Score ___/ 10

Sensor Adhesion Over Time:

How well did the sensors remain attached throughout the wear period? (10 is very well adhered, 1 is very poorly adhered)

First application (applied without constraints)

Adhesive: Toupee Tape

Hand worn (circle one): Left / Right

Score ___/ 10

Adhesive: Derma-tac

Hand worn (circle one): Left / Right

Score ___/ 10

Second application (applied with constraints)

Adhesive: Toupee Tape

Hand worn (circle one): Left / Right

Score ___/ 10

Adhesive: Derma-tac

Hand worn (circle one): Left / Right

Score ___/ 10

Final application (applied without constraints)

Adhesive: Toupee Tape

Hand worn (circle one): Left / Right

Score ___/ 10

Adhesive: Derma-tac

Hand worn (circle one): Left / Right

Score ___/ 10

Wiring Management:

How effective was the wiring layout in minimizing obstruction or discomfort? (10 is very effective, 1 is very ineffective)

First application (applied without constraints)

Adhesive: Toupee Tape

Hand worn (circle one): Left / Right

Score ___/ 10

Adhesive: Derma-tac

Hand worn (circle one): Left / Right

Score ___/ 10

Second application (applied with constraints)

Adhesive: Toupee Tape

Hand worn (circle one): Left / Right

Score ___/ 10

Adhesive: Derma-tac

Hand worn (circle one): Left / Right

Score ___/ 10

Final application (applied without constraints)

Adhesive: Toupee Tape

Hand worn (circle one): Left / Right

Score ___/ 10

Adhesive: Derma-tac

Hand worn (circle one): Left / Right

Score ___/ 10

Overall Experience:

How would you rate your overall experience with the device (10 is excellent, 1 is very poor)?

First application (applied without constraints)

Adhesive: Toupee Tape

Hand worn (circle one): Left / Right

Score ___/ 10

Adhesive: Derma-tac

Hand worn (circle one): Left / Right

Score ___/ 10

Second application (applied with constraints)

Adhesive: Toupee Tape

Hand worn (circle one): Left / Right

Score ___/ 10

Adhesive: Derma-tac

Hand worn (circle one): Left / Right

Score ___/ 10

Final application (applied without constraints)

Adhesive: Toupee Tape

Hand worn (circle one): Left / Right

Score ___/ 10

Adhesive: Derma-tac

Hand worn (circle one): Left / Right

Score ___/ 10

Section 3: Yes/No Questions

Did any of the components detach or become loose during use? If yes, please describe (which hand, first / second / final application, which adhesive method).

☐ Yes ☐ No

Did you experience any skin irritation or discomfort due to the adhesives? If yes, please describe (which hand, first/second/final application, which adhesive method).

☐ Yes ☐ No

Did any part of the device interfere with your normal hand function? If yes, please describe (which hand, first/second/final application, which adhesive method).

☐ Yes ☐ No

Section 4: Participant Feedback

What were the most uncomfortable aspects of wearing the device?

Did you notice any issues with connectivity or sensor stability?

Were there any tasks during which the device felt particularly intrusive or restrictive?

Which adhesive and application method did you prefer and why?

What suggestions do you have to improve the device's comfort, stability, or ease of use?

Any additional comments or concerns?

2. Test administration note taking sheet

Wearable for Continuous Monitoring of Hand Kinematics: Device Performance Test Administration Sheet

Participant ID: ____________________

Participant Dominant Hand: R / L

Date: ____________________

Researcher Initials: ________________

Session Start Time: __________

Session End Time: __________

LH Thumb Measurement: __________

LH Index Measurement: __________

RH Thumb Measurement: __________

RH Index Measurement: __________

LH Index Sensor #: __________

LH Thumb Sensor #: __________

RH Index Sensor #: __________

RH Thumb Sensor #: __________

1. Device Application / Removal Timing

Component	Hand Applied To (R / L)	Application/ Removal Time (Start-End)	Sensor Number	Notes / Photo Number	LED Connectivity
(APP1) Derma-tac Mounted Sensors w/ IMU Wristwatch	R		Thumb:<br><br>Index:		
(APP1) Toupee Tape Mounted Sensors w/ IMU Wristwatch	L		Thumb:<br><br>Index:		
(REM1) Derma-tac Removal Sensors w/ IMU Wristwatch			Thumb:<br><br>Index:		
(REM1) Toupee Tape Removal Sensors w/ IMU Wristwatch			Thumb:<br><br>Index:		
(APP2) Constrained DT Application			Thumb:<br><br>Index:		
(APP2)			Thumb:		

**Constrained TT Application**			**Index:**		
**(REM2) Constrained DT Removal**			**Thumb:**<br><br>**Index:**		
**(REM2) Constrained TT Removal**			**Thumb:**<br><br>**Index:**		
**(APP3) Final DT Application**			**Thumb:**<br><br>**Index:**		
**(APP3) Final TT Application**			**Thumb:**<br><br>**Index:**		
**(REM3) Final DT Removal**			**Thumb:**<br><br>**Index:**		
**(REM3) Final TT Removal**			**Thumb:**<br><br>**Index:**		

2. Fine Motor Dexterity Tasks

Task	Trial	Start - End Time	Sensor Observations / Movement	Photos	LED Connectivity
Pinch & Grasp Test	1 - Left (TT)				
	2 - Right (DT)				
Writing Simulation	1 - Left (TT)				
	2 - Right (DT)				
Typing Simulation	1				
Rotational Movement (Wrist)	1 - Left (TT)				
	2 - Right (DT)				

3. Simulated Activities of Daily Living (ADLs)

Task	Trial	Start - End Time	RH - DT Observations	LH - TT Observations	Photos	LED Connectivity
Eating Simulation	1					
Phone Interaction	1					
Wet Testing	1					
Dressing - Putting on	1					

**Dressing - Taking off**	**1**					

4. Extended Wear Period

Start Time	End Time	Issues Reported During Period (circle all that apply)	Note + Photo #	LED Connectivity
		Sensor Shift / Discomfort / Wiring Detachment / Adhesion Loss		

5. Additional Comments
